# Synthesis and *in vitro* and *in vivo* anti-inflammatory activity of novel 4-ferrocenylchroman-2-one derivatives

**DOI:** 10.1080/14756366.2019.1664499

**Published:** 2019-09-17

**Authors:** Wei-Yun Guo, Liu-Zeng Chen, Bang-Nian Shen, Xin-Hua Liu, Guang-Ping Tai, Qing-Shan Li, Li Gao, Ban-Feng Ruan

**Affiliations:** a School of Food and Biological Engineering, Hefei University of Technology, Hefei, PR China;; b School of Pharmacy, Anhui Province Key Laboratory of Major Autoimmune Diseases, Anhui Medical University, Hefei, PR China;; c Key Lab of Biofabrication of Anhui Higher Education Institution Centre for Advanced Biofabrication, Hefei University, Hefei, PR China

**Keywords:** Ferrocene, chroman-2-one, design, synthesis, anti-inflammatory activity

## Abstract

A series of novel 4-ferrocenylchroman-2-one derivatives were designed and synthesised to discover potent anti-inflammatory agents for treatment of arthritis. All the target compounds had been screened for their anti-inflammatory activity by evaluating the inhibition effect of LPS-induced NO production in RAW 264.7 macrophages. Among them, 4-ferrocenyl-3,4-dihydro-2H-benzo[*g*]chromen-2-one (**3h**) was found to be the most potent compound in inhibiting the productions of NO with low toxicity. This compound also exhibited significant inhibition of the productions of IL-6 and TNF-α in RAW 264.7 macrophages. Preliminary mechanism studies indicated that compound **3h** could inhibit the activation of LPS-induced NF-κB and MAPKs signalling pathways. The *in vivo* anti-inflammatory effect of this compound was determined in the rat adjuvant-induced arthritis model.

## Introduction

Rheumatoid arthritis (RA), which affects about 1% of the population worldwide, is a chronic systemic inflammatory disease characterised by inflammation and progressive joint destruction^1^^,^^2^. The clinical therapies to date mainly focused on nonsteroidal anti-inflammatory drugs (NSAIDs), disease-modifying antirheumatic drugs (DMARDs), and biological products like TNF-α antibody infliximab, the soluble TNF-α receptor etanercept, and interleukin-1 (IL-1)-receptor antagonist anakinra^3^^,^^4^. The unknown cause and doubtful prognosis make the disease more complicated. Moreover, the current conventional and biological therapies for RA give only partial responses^5^. There is still a critical need for discovering novel agents with different mechanisms of action for patients unresponsive to current therapies.

**Figure 1.**
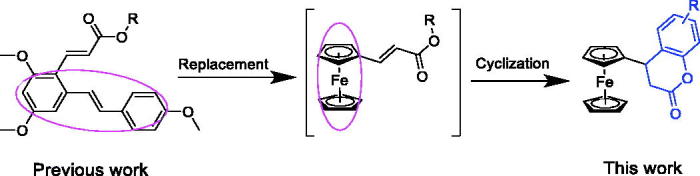
The continuous workflow.

The pathologic lesions of RA are driven, in part, by the production of inflammatory mediators in synoviocytes and macrophages, likely involving the transcription factor nuclear factor κB (NF-κB)^6^. In a previous paper, we reported a resveratrol-based cinnamic ester hybridbenzo[*d*] [*1,3*] dioxol-5-yl (*E*)-3-(2,4-dimethoxy-6-((*E*)-4-methoxystyryl) phenyl) acrylate (**A**, Figure 1), this compound exhibited potent inhibitory effect against LPS-stimulated NO production as well as the expressions of iNOS and COX-2 in RAW 264.7 cells^7^. Furthermore, this compound could exert the anti-inflammatory activity partly due to its inhibitory effect on the NF-κB signalling pathway. As our continuous work to discover novel anti-inflammatory agents^8^, we have made some modifications to this compound. First, given that ferrocene and its derivatives usually are robust, lipophilic, nontoxic, and have good redox properties^9^, the resveratrol fragment of compound **A** was replaced by ferrocene moiety to afford intermediate **B**. Then, the acrylate group was cyclised to the corresponding compounds those bearing chromen-2-one rings (**3a**–**3s**). We then preliminarily evaluated their anti-inflammatory activities by evaluating their inhibitory effects against LPS-stimulated NO, IL-6, and TNF-α production in RAW 264.7 cells. The results indicated that when the substituted groups were naphthalenyl and benzodioxol, the corresponding compounds (**3h**–**3j**) exhibited the most potent anti-inflammatory activities. Among them, compound **3h** was selected to carry out further *in vitro* and *in vivo* evaluations for exploration of the possible anti-inflammatory mechanisms.

## Materials and methods

### General

All commercially available reagents and solvents were purchased and used without further purification. Reactions were monitored by analytical thin-layer chromatography (TLC) and visualised under UV light (*λ* = 254 or 365 nm). Purification by chromatography column was carried on by using silica gel (200–300 mesh). ^1^H and ^13^C NMR (nuclear magnetic resonance) spectra were recorded using an Agilent 600 MHz spectrometer (Agilent Technologies, Palo Alto, CA). The ESI-MS spectra were recorded on a Mariner System 5304 Mass spectrometer. Melting points were determined on a XT4MP apparatus (Taike Corp., Beijing, China), and are uncorrected.

### Chemistry

#### Synthesis of compound (2)

A mixture of ferrocenecarboxaldehyde **1** (2.14 g, 10 mmol) and malonic acid (1.22 g, 12 mmol) in pyridine (30 mL) was stirred at room temperature for 30 min, then 0.2 mL of piperidine were added dropwise, followed by stirring at 95 °C for 4 h^10^. The reaction was monitored by TLC. After the reaction was completed, the mixture was poured into dilute acid and extracted with ethyl acetate. The organic phase was collected, dried, and evaporated under reduced pressure to afford the crude product. After purifying by column chromatography on silica gel (ethyl acetate/petroleum ether = 1:3), compound **2** was obtained as yellow solid. Yield: 79%. ^1^H NMR (600 MHz, CDCl_3_): *δ* 7.72–7.69 (*d*, *J* = 18.0 Hz, 1H), 6.05–6.03 (*d*, *J* = 12.0 Hz, 1H), 4.52 (s, 2H), 4.45 (s, 2H), and 4.17 (s, 5H).

#### 4-Ferrocenyl-7-methylchroman-2-one (3a)

A mixture of **2** (0.26 g, 1.0 mmol) and *m*-cresol (0.13 g, 1.2 mmol) in TFA (3 mL) was stirred at room temperature for 2 h^11^. After complete consumption of starting material (TLC monitoring), the reaction mixture was diluted with 20 mL CH_2_Cl_2_. The organic layer was washed with water (40 mL) twice, dried over Na_2_SO_4_, and concentrated under reduced pressure. The residue was purified via column chromatography on SiO_2_ to afford **3a**. Pale yellow solid, yield: 63%, Mp: 138.5–140.6 °C. ^1^H NMR (600 MHz, CDCl_3_): *δ* 7.02 (*d*, *J* = 7.8 Hz, 1H), 6.90 (*d*, *J* = 7.2 Hz, 1H), 6.87 (s, 1H), 4.15 (s, 7H), 4.05 (s, 1H), 4.03 (s, 1H), 3.99 (t, *J* = 6 Hz, 1H), 3.11 (dd, *J_1_* =*J_2_=* 5.4 Hz, 1H), 3.01 (dd, *J_1_*=*J_2_=*6 Hz, 1H), 2.33 (s, 3H). ^13^CNMR (151 MHz, CDCl_3_): *δ* 168.6 (s), 152.3 (s), 150.1 (s), 127.3 (s), 124.2 (s), 123.4 (s), 121.2 (s), 114.0 (s), 89.2 (s), 68.7 (s), 67.9 (s), 65.3 (s), 36.0 (s), 34.7 (s), and 31.2 (s). MS (ESI): 346.1 (C_20_H_18_FeO_2_, [M + H^+^]).

#### 4-Ferrocenyl-7-ethylchroman-2-one (3b)

A mixture of **2** (0.26 g, 1.0 mmol) and 3-ethylphenol (0.15 g, 1.2 mmol) in TFA (3 mL) was stirred at room temperature for 2 h. After complete consumption of starting material (TLC monitoring), the reaction mixture was diluted with 20 mL CH_2_Cl_2_. The organic layer was washed with water (40 mL) twice, dried over Na_2_SO_4_, and concentrated under reduced pressure. The residue was purified via column chromatography on SiO_2_ to afford **3b**. Oil, yield: 73%. ^1^H NMR (600 MHz, CDCl_3_): *δ* 7.04 (d, *J* = 7.8 Hz, 1H), 6.92 (d, *J* = 7.8 Hz, 1H), 6.90 (s, 1H), 4.16 (s, 7H), 4.05 (d, *J* = 17.4 Hz, 2H), 4.00 (t, *J* = 6 Hz, 1H), 3.12 (dd, *J_1_* = 6 Hz, *J_2_* 5.4 Hz, 1H), 3.02 (dd, *J_1_* = *J_2_=*6 Hz, 1H), 2.63 (q, *J* = 7.8 Hz, 2H), 1.22 (t, *J* = 7.2 Hz, 3H). ^13^CNMR (151 MHz, CDCl_3_): *δ* 168.6 (s), 150.9 (s), 145.1 (s), 127.6 (s), 123.9 (s), 116.2 (s), 89.2 (s), 68.7 (s), 68.2 (s), 67.9 (s), 65.3 (s), 36.1 (s), 34.7 (s), 28.3 (s), and 15.3 (s). MS (ESI): 361.2 (C_21_H_20_FeO_2_, [M + H]^+^).

#### 4-Cyclohexyl-6-pentylchroman-2-one (3c)

A mixture of **2** (0.26 g, 1.0 mmol) and 4-pentylphenol (0.20 g, 1.2 mmol) in TFA (3 mL) was stirred at room temperature for 2 h. After complete consumption of starting material (TLC monitoring), the reaction mixture was diluted with 20 mL CH_2_Cl_2_. The organic layer was washed with water (40 mL) twice, dried over Na_2_SO_4_, and concentrated under reduced pressure. The residue was purified via column chromatography on SiO_2_ to afford **3c**. Oil, yield: 65%. ^1^H NMR (600 MHz, CDCl_3_): *δ* 7.04 (d, *J* = 7.8 Hz, 1H), 6.96 (d, *J* = 8.4 Hz, 1H), 6.75 (s, 1H), 4.22 (s, 7H), 4.14 (s, 2H), 3.97 (s, 1H), 3.09 (d, *J* = 13.8 Hz, 1H), 3.00 (d, *J* = 15 Hz, 1H), 2.54 (t, *J* = 7.2 Hz, 2H), 1.31 (m, 6H), 0.89 (t, *J* = 7.8 Hz, 3H). ^13^C NMR (151 MHz, CDCl_3_): *δ* 168.6 (s), 153.6 (s), 148.9 (s), 139.0 (s), 135.0 (s), 129.3 (s), 128.2 (s), 116.6 (s), 114.9 (s), 69.3 (s), 68.7 (s), 68.2 (s), 66.1 (s), 36.0 (s), 35.1 (s), 31.3 (s), 22.4 (s), and 13.9 (s). MS (ESI): 402.1 (C_24_H_26_FeO_2_, [M + H]^+^).

#### 6-(Tert-butyl)-4-cyclohexylchroman-2-one (3d)

A mixture of **2** (0.26 g, 1.0 mmol) and 4-*tert*-butylphenol (0.18 g, 1.2 mmol) in TFA (3 mL) was stirred at room temperature for 2 h. After complete consumption of starting material (TLC monitoring), the reaction mixture was diluted with 20 mL CH_2_Cl_2_. The organic layer was washed with water (40 mL) twice, dried over Na_2_SO_4_, and concentrated under reduced pressure. The residue was purified via column chromatography on SiO_2_ to afford **3d**. Pale yellow solid, yield: 81%, Mp: 187.5–188.4 °C. ^1^H NMR (600 MHz, CDCl_3_): *δ* 7.29 (dd, *J_1_*=1.8 Hz, *J_2_* =8.4 Hz, 1H), 7.21 (d, *J* = 1.8 Hz, 1H), 7.00 (d, *J* = 8.4 Hz, 1H), 4.14 (m, 7H), 4.05 (s, 1H), 4.02 (d*, J*= 5.4 Hz, 1H), 4.00 (s, 1H), 3.09 (dd, *J_1_* = *J_2_* = 6 Hz, 1H), 3.02 (dd, *J_1_*= 5.4 Hz, *J_2_*= 4.8 Hz, 1H), and 1.31 (s, 9H). ^13^C NMR (151 MHz, CDCl_3_): *δ* 168.5 (s), 148.8 (s), 147.4 (s), 125.5 (s), 124.9 (s), 116.4 (s), 89.4 (s), 68.7 (s), 68.2 (s), 68.1 (s), 67.8 (s), 67.5 (s), 65. 6 (s), 36.4 (s), 35.4 (s), 34.4 (s), and 31.4 (s). MS (ESI): 388.3 (C_23_H_24_FeO_2_, [M + H]^+^).

#### 7-(Tert-butyl)-4-cyclohexylchroman-2-one (3e)

A mixture of **2** (0.26 g, 1.0 mmol) and 3-*tert*-butylphenol (0.18 g, 1.2 mmol) in TFA (3 mL) was stirred at room temperature for 2 h. After complete consumption of starting material (TLC monitoring), the reaction mixture was diluted with 20 mL CH_2_Cl_2_. The organic layer was washed with water (40 mL) twice, dried over Na_2_SO_4_, and concentrated under reduced pressure. The residue was purified via column chromatography on SiO_2_ to afford **3e**. Pale yellow solid, yield: 77%, Mp: 191.2–192.6 °C. ^1^H NMR (600 MHz, CDCl_3_): *δ* 7.10 (s, 1H), 7.07 (s, 2H), 4.16 (s, 7H), 4.09 (s, 1H), 4.04 (s, 1H), 4.00 (t, *J* = 6 Hz, 1H), 3.14 (dd, *J_1_* = *J_2_ =* 6 Hz, 1H), 3.03 (dd, *J_1_*=*J_2_=*6.6 Hz, 1H), and 1.29 (s, 9H). ^13^C NMR (151 MHz, CDCl_3_): *δ* 168.4 (s), 147.2 (s), 145.4 (s), 144.2 (s), 124.2 (s), 118.9 (s), 106.9 (s), 101.6 (s), 99.0 (s), 89.0 (s), 68.7 (s), 67.9 (s), 65.2 (s), 35.8 (s), and 34.9 (s). MS (ESI): 388.3 (C_23_H_24_FeO_2_, [M + H]^+^).

#### 4-Ferrocenyl-6-(2,4,4-trimethylpentan-2-yl)chroman-2-one (3f)

A mixture of **2** (0.26 g, 1.0 mmol) and 4-*tert*-octylphenol (0.25 g, 1.2 mmol) in TFA (3 mL) was stirred at room temperature for 2 h. After complete consumption of starting material (TLC monitoring), the reaction mixture was diluted with 20 mL CH_2_Cl_2_. The organic layer was washed with water (40 mL) twice, dried over Na_2_SO_4_, and concentrated under reduced pressure. The residue was purified via column chromatography on SiO_2_ to afford **3f**. Oil, yield: 74%. ^1^H NMR (600 MHz, CDCl_3_): *δ* 7.26 (s, 1H), 7.16 (s, 1H), 6.97 (d, *J* = 8.4 Hz, 1H), 4.13 (m, 7H), 4.02 (m, 3H), 3.09 (dd, *J_1_* = 6 Hz, *J_2_ =* 5.4 Hz, 1H), 3.03 (dd, *J_1_* = 4.2 Hz, *J_2_=*4.8 Hz, 1H), 1.71 (s, 2H), 1.34 (s, 6H), and 0.72 (s, 9H). ^13^C NMR (151 MHz, CDCl_3_): *δ* 168.6 (s), 148.6 (s), 146.4 (s), 126.1 (s), 125.6 (s), 125.1 (s), 116.1 (s), 89.3 (s), 68.7 (s), 67.7 (s), 65.4 (s), 56.8 (s), 38.3 (s), 36.1 (s), 35.3 (s), 32.3 (s), 31.7 (s), and 31.5 (s). MS (ESI): 444.4 (C_27_H_32_FeO_2_, [M + H]^+^).

#### 6-Cyclohexyl-4-ferrocenylchroman-2-one (3g)

A mixture of **2** (0.26 g, 1.0 mmol) and 4-cyclohexylphenol (0.21 g, 1.2 mmol) in TFA (3 mL) was stirred at room temperature for 2 h. After complete consumption of starting material (TLC monitoring), the reaction mixture was diluted with 20 mL CH_2_Cl_2_. The organic layer was washed with water (40 mL) twice, dried over Na_2_SO_4_, and concentrated under reduced pressure. The residue was purified via column chromatography on SiO_2_ to afford **3g**. Pale yellow solid, yield: 65%, Mp: 146.0–147.0 °C. ^1^H NMR (600 MHz, CDCl_3_): *δ* 7.09 (d, *J* = 8.4 Hz, 1H), 7.00 (s, 1H), 6.98 (d, *J* = 7.8 Hz, 1H), 4.17 (s, 7H), 4.08 (d, *J* = 14.4 Hz, 2H), 3.98 (t, *J* = 6 Hz, 1H), 3.09 (dd, *J_1_* = 5.4 Hz, *J_2_ =* 4.8 Hz, 1H), 3.00 (dd, *J_1_* = 5.4 Hz, *J_2_ =* 4.8 Hz, 1H), 2.46 (m, 1H), 1.84 (m, 4H), 1.37 (m, 4H), 1.26 (m, 2H). ^13^C NMR (151 MHz, CDCl_3_): *δ* 168.5 (s), 149.1 (s), 144.3 (s), 126.3 (s), 125.9 (s), 116.7 (s), 69.0 (s), 68.4 (s), 68.0 (s), 67.9 (s), 65.8 (s), 43.9 (s), 36.3 (s), 35.3 (s), 34.6 (s), 26.7 (s), and 26.0 (s). MS (ESI): 414.3 (C_25_H_26_FeO_2_, [M + H]^+^).

#### 4-Ferrocenyl-3,4-dihydro-2H-benzo[g]chromen-2-one (3h)

A mixture of **2** (0.26 g, 1.0 mmol) and 2-naphthol (0.17 g, 1.2 mmol) in TFA (3 mL) was stirred at room temperature for 2 h. After complete consumption of starting material (TLC monitoring), the reaction mixture was diluted with 20 mL CH_2_Cl_2_. The organic layer was washed with water (40 mL) twice, dried over Na_2_SO_4_, and concentrated under reduced pressure. The residue was purified via column chromatography on SiO_2_ to afford **3h**. Yellow needle-like solid, yield: 70%, Mp: 159.0–161.0 °C. ^1^H NMR (600 MHz, CDCl_3_): *δ* 8.09 (d, *J* = 8.4 Hz, 1H), 7.86 (d, *J* = 7.8 Hz, 1H), 7.78 (d, *J* = 8.4 Hz, 1H), 7.62 (t, *J* = 8.4 Hz, 1H), 7.48 (t, *J* = 7.8 Hz, 1H), 7.27 (d, *J* = 8.4 Hz, 1H), 4.67 (d, *J* = 6.6 Hz, 1H), 4.14 (s, 7H), 4.04 (s, 1H), 3.41 (d, *J* = 16.2 Hz, 1H), and 3.08 (dd, *J_1_* = *J_2_ =* 6.6 Hz, 1H). ^13^C NMR (151 MHz, CDCl_3_): *δ* 168.5 (s), 148.5 (s), 131.1 (s), 130.7 (s), 129.3 (s), 128.8 (s), 127.0 (s), 125.0 (s), 123.3 (s), 119.7 (s), 117.7 (s), 90.0 (s), 68.9 (s), 67.8 (s), 66.9 (s), 35.3 (s), and 30.9 (s). MS (ESI): 383.1 (C_23_H_18_FeO_2_, [M + H]^+^).

#### 4-Ferrocenyl-3,4-dihydro-2H-benzo[h]chromen-2-one (3i)

A mixture of **2** (0.26 g, 1.0 mmol) and 1-naphthol (0.17 g, 1.2 mmol) in TFA (3 mL) was stirred at room temperature for 2 h. After complete consumption of starting material (TLC monitoring), the reaction mixture was diluted with 20 mL CH_2_Cl_2_. The organic layer was washed with water (40 mL) twice, dried over Na_2_SO_4_, and concentrated under reduced pressure. The residue was purified via column chromatography on SiO_2_ to afford **3i**. Yellow needle-like solid, yield: 68%, Mp: 162.0–164.0 °C. ^1^H NMR (600 MHz, CDCl_3_): *δ* 8.27 (d, *J* = 8.4 Hz, 1H), 7.82 (d, *J* = 7.8 Hz, 1H), 7.60 (d, *J* = 7.8 Hz, 1H), 7.54 (m, 2H), 7.29 (d, *J* = 8.4 Hz, 1H), 4.16 (m, 7H), 4.08 (m, 2H), 3.23 (dd, *J_1_* = *J_2_* = 6 Hz, 1H), and 3.17 (dd, *J_1_*= 4.8 Hz, *J_2_* = 5.4 Hz, 1H). ^13^C NMR (151 MHz, CDCl_3_): *δ 1*70.8 (s), 136.2 (s), 130.2 (s), 129.3 (s), 127.6 (s), 126.9 (s), 126.6 (s), 126.3 (s), 123.9 (s), 123.5 (s), 92.0 (s), 71.4 (s), 71.0 (s), 70.5 (s), 68.2 (s), 38.9 (s), and 38.1 (s). MS (ESI): 383.1 (C_23_H_18_FeO_2_, [M + H]^+^).

#### 8-Ferrocenyl-7,8-dihydro-6H-[1,3]dioxolo[4,5-g]chromen-6-one (3j)

A mixture of **2** (0.26 g, 1.0 mmol) and 1,3-benzodioxol-5-ol (0.17 g, 1.2 mmol) in TFA (3 mL) was stirred at room temperature for 2 h. After complete consumption of starting material (TLC monitoring), the reaction mixture was diluted with 20 mL CH_2_Cl_2_. The organic layer was washed with water (40 mL) twice, dried over Na_2_SO_4_, and concentrated under reduced pressure. The residue was purified via column chromatography on SiO_2_ to afford **3j**. Yellow needle-like solid, yield: 81%, Mp: 162.7–168.1 °C. ^1^H NMR (600 MHz, CDCl_3_): *δ* 6.58 (s, 1H), 6.55 (s, 1H), 5.94 (s, 1H), 5.92 (s, 1H), 4.16 (s, 7H), 4.07 (s, 1H), 4.02 (s, 1H), 3.91 (t, *J* = 6 Hz, 1H), 3.11 (dd, *J_1_*= 5.4 Hz, *J_2_ =* 6 Hz, 1H), and 2.99 (dd, *J_1_* = 6.6 Hz, *J_2_ =* 6 Hz, 1H). ^13^C NMR (151 MHz, CDCl_3_): *δ* 168.6 (s), 150.8 (s), 138.7 (s), 127.5 (s), 125.1 (s), 124.2 (s), 123.4 (s), 117.3 (s), 89.2 (s), 66.7 (s), 68.2 (s), 67.8 (s), 65.4 (s), 36.2 (s), 34.7 (s), and 21.0 (s). MS (ESI):376.2 (C_20_H_16_FeO_4_, [M + Na]^+^).

#### 6,8-Dimethyl-4-ferrocenylchroman-2-one (3k)

A mixture of **2** (0.26 g, 1.0 mmol) and 2,4-dimethylphenol (0.15 g, 1.2 mmol) in TFA (3 mL) was stirred at room temperature for 2 h. After complete consumption of starting material (TLC monitoring), the reaction mixture was diluted with 20 mL CH_2_Cl_2_. The organic layer was washed with water (40 mL) twice, dried over Na_2_SO_4_, and concentrated under reduced pressure. The residue was purified via column chromatography on SiO_2_ to afford **3k**. Yellow needle-like solid, Yield: 83%, Mp: 155.0–157.0 °C. ^1^H NMR (600 MHz, CDCl_3_): *δ* 6.91 (s, 1H), 6.76 (s, 1H), 4.15 (s, 7H), 4.08 (s, 1H), 4.05 (s, 1H), 3.96 (t, *J* = 6 Hz, 1H), 3.10 (dd, *J_1_* = 6 Hz, *J_2_ =* 5.4 Hz, 1H), 3.02 (dd, *J_1_* = *J_2_ =* 6 Hz, 1H), 2.27 (s, 3H), and 2.26 (s, 3H). ^13^C NMR (151 MHz, CDCl_3_): *δ* 168.7 (s), 147.0 (s), 133.3 (s), 130.5 (s), 125.9 (s), 125.6 (s), 89.2 (s), 68.7 (s), 68.1 (s), 67.9 (s), 65.4 (s), 36.0 (s), 35.2 (s), 20.7 (s), and 15.6 (s). MS (ESI): 399.0 (C_21_H_20_FeO_2_, [M + K]^+^).

#### 7,8-Dimethyl-4-ferrocenylchroman-2-one (3l)

A mixture of **2** (0.26 g, 1.0 mmol) and 2,3-dimethylphenol (0.15 g, 1.2 mmol) in TFA (3 mL) was stirred at room temperature for 2 h. After complete consumption of starting material (TLC monitoring), the reaction mixture was diluted with 20 mL CH_2_Cl_2_. The organic layer was washed with water (40 mL) twice, dried over Na_2_SO_4_, and concentrated under reduced pressure. The residue was purified via column chromatography on SiO_2_ to afford **3l**. Yellow solid, yield: 73%, Mp: 151.0–153.0 °C. ^1^H NMR (600 MHz, CDCl_3_): *δ* 6.89 (d, *J* = 7.2 Hz, 1H), 6.85 (d, *J* = 7.2 Hz, 1H), 4.20 (m, 7H), 4.11 (s, 2H), 3.97 (t, *J* = 6 Hz, 1H), 3.08 (dd, *J_1_* = 4.8 Hz, *J_2_ =* 4.2 Hz, 1H), 3.00 (dd, *J_1_* = *J_2_ =* 4.2 Hz, 1H), 2.27 (s, 3H), and 2.22 (s, 3H). ^13^C NMR (151 MHz, CDCl_3_): *δ* 168.9 (s), 153.6 (s), 148.9 (s), 137.3 (s), 125.9 (s), 124.7 (s), 124.4 (s), 123.8 (s), 122.2 (s), 112.5 (s), 89.6 (s), 69.0 (s), 68.4 (s), 65.7 (s), 35.9 (s), 35.1 (s), 19.8 (s), and 11.7 (s). MS (ESI): 362.3 (C_21_H_20_FeO_2_, [M + H]^+^).

#### 6,7-Dimethyl-4-ferrocenylchroman-2-one (3m)

A mixture of **2** (0.26 g, 1.0 mmol) and 3,4-dimethylphenol (0.15 g, 1.2 mmol) in TFA (3 mL) was stirred at room temperature for 2 h. After complete consumption of starting material (TLC monitoring), the reaction mixture was diluted with 20 mL CH_2_Cl_2_. The organic layer was washed with water (40 mL) twice, dried over Na_2_SO_4_, and concentrated under reduced pressure. The residue was purified via column chromatography on SiO_2_ to afford **3m**. Yellow needle solid, yield: 76%, Mp: 157.0–159.0 °C. ^1^H NMR (600 MHz, CDCl_3_): *δ* 6.88 (s, 1H), 6.83 (s, 1H), 4.15 (s, 7H), 4.07 (s, 1H), 4.03 (s, 1H), 3.96 (t, *J* = 6 Hz, 1H), 3.10 (dd, *J_1_* = *J_2_ =* 6 Hz, 1H), 3.01 (dd, *J_1_* = *J_2_ =* 6 Hz, 1H), 2.22 (s, 3H), and 2.20 (s, 3H). ^13^C NMR (151 MHz, CDCl_3_): *δ* 168.7 (s), 148.8 (s), 137.0 (s), 132.5 (s), 128.6 (s), 128.5 (s), 123.3 (s), 117.7 (s), 89.4 (s), 68.7 (s), 65.3 (s), 36.2 (s), 34.7 (s), 19.5 (s), and 19.1 (s). MS (ESI): 383.1 (C_21_H_20_FeO_2_, [M + Na]^+^).

#### 5,7-Dimethyl-4-ferrocenylchroman-2-one (3n)

A mixture of **2** (0.26 g, 1.0 mmol) and 3,5-dimethylphenol (0.15 g, 1.2 mmol) in TFA (3 mL) was stirred at room temperature for 2 h. After complete consumption of starting material (TLC monitoring), the reaction mixture was diluted with 20 mL CH_2_Cl_2_. The organic layer was washed with water (40 mL) twice, dried over Na_2_SO_4_, and concentrated under reduced pressure. The residue was purified via column chromatography on SiO_2_ to afford **3n**. Yellow needle solid, yield: 66%, Mp: 116.6–120.0 °C. ^1^H NMR (600 MHz, CDCl_3_): *δ* 6.78 (s, 1H), 6.73 (s, 1H), 4.15 (s, 5H), 4.11 (*d*, *J* = 7.8 Hz, 2H), 4.08 (s, 1H), 4.04 (d, *J* = 6.6 Hz 1H), 4.00 (s, 1H), 3.32 (d, *J* = 16.2 Hz, 1H), 2.94 (dd, *J_1_* = *J_2_ =* 6.6 Hz, 1H), 2.38 (s, 3H), 2.28 (s, 3H). ^13^C NMR (151 MHz, CDCl_3_): *δ* 169.1 (s), 151.0 (s), 138.1 (s), 135.5 (s), 127.3 (s), 122.1 (s), 115.5 (s), 90.4 (s), 68.9 (s), 67.9 (s), 67.4 (s), 65.8 (s), 36.1 (s), 31.4 (s), 20.9 (s), and 18.9 (s). MS (ESI): 383.1 (C_21_H_20_FeO_2_, [M + Na]^+^).

#### 5,7,8-Trimethyl-4-ferrocenylchroman-2-one (3o)

A mixture of **2** (0.26 g, 1.0 mmol) and 2,3,5-dimethylphenol (0.16 g, 1.2 mmol) in TFA (3 mL) was stirred at room temperature for 2 h. After complete consumption of starting material (TLC monitoring), the reaction mixture was diluted with 20 mL CH_2_Cl_2_. The organic layer was washed with water (40 mL) twice, dried over Na_2_SO_4_, and concentrated under reduced pressure. The residue was purified via column chromatography on SiO_2_ to afford **3o**. Yellow needle-like solid, Yield: 77%, Mp: 188.6–191.6 °C. ^1^H NMR (600 MHz, CDCl_3_): *δ* 6.79 (s, 1H), 4.19 (m, 7H), 4.10 (s, 3H), 3.29 (d, *J* = 7.2 Hz, 1H), 2.90 (dd, *J_1_* = *J_2_ =*6 Hz, 1H), 2.38 (s, 3H), 2.19 (s, 3H), 2.12 (s, 3H). ^13^C NMR (151 MHz, CDCl_3_): *δ* 169.4 (s), 153.4 (s), 149.0 (s), 137.9 (s), 136.7 (s), 131.8 (s), 127.5 (s), 123.1 (s), 119.1 (s), 113.3 (s), 69.1 (s), 68.0 (s), 66.1 (s), 35.8 (s), 20.8 (s), 18.7 (s), and 11.5 (s). MS (ESI): 374.3 (C_22_H_22_FeO_2_, [M + H]^+^).

#### 6-Isopropyl-7-methyl-4-ferrocenylchroman-2-one (3p)

A mixture of **2** (0.26 g, 1.0 mmol) and 3-methyl-4-propan-2-ylphenol (0.18 g, 1.2 mmol) in TFA (3 mL) was stirred at room temperature for 2 h. After complete consumption of starting material (TLC monitoring), the reaction mixture was diluted with 20 mL CH_2_Cl_2_. The organic layer was washed with water (40 mL) twice, dried over Na_2_SO_4_, and concentrated under reduced pressure. The residue was purified via column chromatography on SiO_2_ to afford **3p**. Oil, yield: 83%. ^1^H NMR (600 MHz, CDCl_3_): *δ* 7.01 (s, 1H), 6.82 (s, 1H), 4.24 (m, 7H), 4.17 (s, 1H), 4.14 (s, 1H), 3.95 (s, 1H), 3.09 (m, 1H), 3.04 (*d*, *J* = 15 Hz, 1H), 2.96 (d, *J* = 15 Hz, 1H), 2.31 (s, 3H), and 1.20 (d, *J* = 6.6 Hz, 6H). ^13^C NMR (151 MHz, CDCl_3_): *δ* 168.5 (s), 148.4 (s), 142.9 (s), 135.6 (s), 124.2 (s), 123.3 (s), 118.0 (s), 107.2 (s), 69.5 (s), 36.5 (s), 35.1 (s), 28.7 (s), 23.3 (s), 23.2 (s), and 18.9 (s). MS (ESI):411.3 (C_23_H_24_FeO_2_, [M + Na]^+^).

#### 8-(Tert-butyl)-6-methyl-4-ferrocenylchroman-2-one (3q)

A mixture of **2** (0.26 g, 1.0 mmol) and 2-*tert*-butyl-4-methylphenol (0.20 g, 1.2 mmol) in TFA (3 mL) was stirred at room temperature for 2 h. After complete consumption of starting material (TLC monitoring), the reaction mixture was diluted with 20 mL CH_2_Cl_2_. The organic layer was washed with water (40 mL) twice, dried over Na_2_SO_4_, and concentrated under reduced pressure. The residue was purified via column chromatography on SiO_2_ to afford **3q**. Oil, yield: 86%. ^1^H NMR (600 MHz, CDCl_3_): *δ* 7.07 (s, 1H), 6.79 (s, 1H), 4.18 (s, 7H), 4.09 (d, *J* = 11.4 Hz, 2H), 3.97 (t, *J* = 6 Hz, 1H), 3.11 (dd, *J_1_* = *J_2_ =*5.4 Hz, 1H), 3.00 (dd, *J_1_* = *J_2_ =*6 Hz, 1H), 2.28 (s, 3H), and 1.42 (s, 9H). ^13^C NMR (151 MHz, CDCl_3_): *δ* 168.7 (s), 151.9 (s), 147.4 (s), 137.6 (s), 133.1 (s), 129.3 (s), 116.3 (s), 68.8 (s), 68.3 (s), 68.1 (s), 67.8 (s), 65.6 (s), 35.8 (s), 29.5 (s), and 21.1 (s). MS (ESI): 402.3 (C_24_H_26_FeO_2_, [M + H]^+^).

#### 8-(Tert-butyl)-6-methyl-4-ferrocenylchroman-2-one (3r)

A mixture of **2** (0.26 g, 1.0 mmol) and 2-*tert*-butyl-4-ethylphenol (0.21 g, 1.2 mmol) in TFA (3 mL) was stirred at room temperature for 2 h. After complete consumption of starting material (TLC monitoring), the reaction mixture was diluted with 20 mL CH_2_Cl_2_. The organic layer was washed with water (40 mL) twice, dried over Na_2_SO_4_, and concentrated under reduced pressure. The residue was purified via column chromatography on SiO_2_ to afford **3r**. Oil, yield: 76%. ^1^H NMR (600 MHz, CDCl_3_): *δ* 7.10 (s, 1H), 6.84 (s, 1H), 4.18 (s, 7H), 4.10 (d, *J* = 9 Hz, 2H), 3.98 (t, *J* = 5.4 Hz, 1H), 3.10 (dd, *J_1_* = *J_2_ =*5.4 Hz, 1H), 3.00 (dd, *J_1_* = *J_2_ =*5.4 Hz, 1H), 2.61 (q, *J* = 7.8 Hz, 2H), 1.42 (s, 9H), 1.22 (t, *J* = 9.6 Hz, 3H). ^13^C NMR (151 MHz, CDCl_3_): *δ* 168.7 (s), 152.1 (s), 147.6 (s), 139.5 (s), 137.7 (s), 135.8 (s), 125.8 (s), 116.3 (s), 68.9 (s), 68.3 (s), 67.9 (s), 65.8 (s), 35.9 (s), 34.7 (s), 29.5 (s), 28.5 (s), and 15.9 (s). MS (ESI): 416.1 (C_25_H_28_FeO_2_, [M + H]^+^).

#### 6,8-Di-tert-butyl-4-ferrocenylchroman-2-one (3s)

A mixture of **2** (0.26 g, 1.0 mmol) and 2,4-di*-tert*-butylphenol (0.25 g, 1.2 mmol) in TFA (3 mL) was stirred at room temperature for 2 h. After complete consumption of starting material (TLC monitoring), the reaction mixture was diluted with 20 mL CH_2_Cl_2_. The organic layer was washed with water (40 mL) twice, dried over Na_2_SO_4_, and concentrated under reduced pressure. The residue was purified via column chromatography on SiO_2_ to afford **3s**. Oil, yield: 81%. ^1^H NMR (600 MHz, CDCl_3_): *δ* 7.31 (s, 1H), 7.07 (s, 1H), 4.16 (s, 7H), 4.10 (s, 1H), 4.03 (s, 1H), 3.09 (dd, *J_1_* = *J_2_ =*5.4 Hz, 1H), 3.00 (dd, *J_1_* = 4.8 Hz, *J_2_ =* 4.2 Hz, 1H), 1.43 (s, 9H), 1.32 (s, 9H). ^13^C NMR (151 MHz, CDCl_3_): *δ* 168.7 (s), 151.8 (s), 146.3 (s), 137.2 (s), 135.1 (s), 126.2 (s), 123.4 (s), 122.9 (s), 115.8 (s), 68.8 (s), 68.2 (s), 67.8 (s), 65.8 (s), 36.2 (s), 35.0 (s), 31.4 (s), and 30.6 (s). MS (ESI): 444.2 (C_27_H_32_FeO_2_, [M + H]^+^).

### *In vitro* anti-inflammatory activity

#### Cell culture

RAW 264.7 cells were obtained from the Cell Bank of the Chinese Academy of Sciences (Shanghai, China). The cells were grown in Dulbecco’s modified Eagle’s medium containing 10% (*v/v*) foetal calf serum (Hyclone, Logan, UT), 100 U/mL penicillin, and 100 mg/mL streptomycin and incubated in a humidified atmosphere of 5% CO_2_ and 95% air at 37 °C.

#### Assay for NO production

RAW 264.7 cells were seeded at a density of 7 × 10^4^ cells per well in 24-well plate with 500 μL of culture medium and incubated for 24 h. The cells were then pretreated with compound **3h** for 1 h before stimulation with LPS (1 μg/mL) for 24 h. NO synthesis was spectrophotometrically determined by assaying the culture supernatants for nitrite using the Griess reagent (Beyotime Biotechnology, Haimen, China). Absorbance was measured at 540 nm and NO concentration was determined using sodium nitrite as a standard.

#### Cytokine analysis

RAW 264.7 cells were pretreated with compound **3h** at concentration of 2.5, 5, and 10 μM for 1 h, then incubated with LPS (0.5 μg/mL) for 24 h. The levels of TNF-α and IL-6 in serum were analysed using ELISA kits (Beyotime, Haimen, China) according to the manufacturer’s instruction. The absorbance was measured with the microplate reader at 450 nm.

#### Cell viability assay (MTT)

RAW 264.7 cells were seeded at a density of 1 × 10^3^ cells per well in 96-well plate for 24 h before treatment. Following treatment with LPS and different concentrations of compound **3h**, cell viability was determined using the MTT assay. Briefly, 10 μL (5 mg/mL) MTT working solution was added to each well and after incubation at 37 °C for 4 h. Then the MTT solution was removed and 200 μL of dimethyl sulphoxide (DMSO) was added to dissolve the crystals. The absorbance of each well at 570 nm was measured using a microplate reader (MQX200, Bio-Tek, Winooski, VT).

#### Western blotting

RAW 264.7 cells were cultured for 24 h and then treated with different concentrations of compound **3h** for 1 h before exposure to LPS. The cells were lysed in 280 μL RIPA cell lysis buffer (Beyotime, Haimen, China) and incubated on ice for 30 min. For Western blotting, proteins were resolved by SDS-PAGE then transferred to PVDF membrane (GE Healthcare, Amersham, UK). The blotted membrane was incubated with specific primary antibody for overnight at 4 °C and further incubated for 1 h with HRP-conjugated secondary antibody.

### *In vivo* anti-inflammatory activity

#### Animals

Healthy male Sprague–Dawley (SD) rats weighing 160–180 g were purchased from Experimental Animal Center of Anhui Medical University (Hefei, China). The animals were cultured under standardised conditions with the humidity of 50–55%, temperature of 23 °C ± 2 °C and have free access to clean food and water. All animal protocols were approved by the Ethics Committee in Animal Experimentation at Hefei University of Technology (Hefei, China) following the guidelines for Care and Use of Laboratory Animals.

#### Establishment of AIA rat

The anti-inflammatory effect of compound **3h** was studied on the complete Freund’s adjuvant (CFA)-induced arthritis (AIA) rat model^12^. Rat AIA was induced by a single intradermal injection of 0.1 mL CFA (10 mg/mL, Sigma, St. Louis, MO) into the right hind paw. Paw swelling and body weight were measured every three days after primary immunisation with CFA to evaluate arthritis.

#### Treatment regimen

Twenty five rats were randomly divided into five groups (five rats per group): (i) the rats received single intradermal injection of 0.1 mL saline (normal), (ii) the AIA rats without treatment (AIA), (iii) the AIA rats received intragastrical administration of aspirin at the dose of 40 mg/kg (aspirin, 40 mg/kg), (iv) the AIA rats received intragastrical administration of compound **3h** at the dose of 40 mg/kg (compound **3h**, 40 mg/kg), and (v) the AIA rats received intragastrical administration of compound **3h** at the dose of 80 mg/kg (compound **3h**, 80 mg/kg).

#### Histopathological examination of joints

The rats were sacrificed by CO_2_ asphyxiation at the end of the experiment. The knee joints were immediately removed, trimmed, fixed in 4% neutral buffered paraformaldehyde solution and then decalcified in 4% HNO_3_ for two days. After decalcification, the tissues were dehydrated in a gradient ethanol series, embedded in paraffin and sectioned at 4 μM thickness. The tissue sections were stained with haematoxylin and eosin (HE) for histopathological examination. The histological images were taken using an Olympus BX51 microscope system (Olympus Corporation, Tokyo, Japan).

### Statistical analysis

Statistical analysis was performed using a one-way ANOVA where *p* value less than .05 was considered statistically significant. Data were expressed as mean ± SEM and analysed using GraphPad Prism version 5.0 (GraphPad Software, Inc., La Jolla, CA). One-way ANOVA with Tukey’s multiple comparison test was used to compare the means of all experimental groups.

## Results and discussion

### Chemistry

**Scheme 1.**
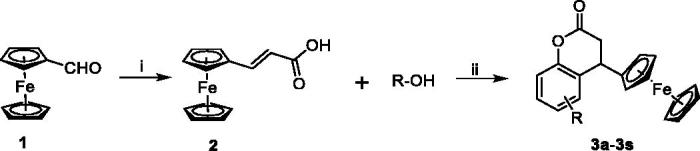
The synthetic route of the target compounds. Reagents and conditions: (i) malonic acid, piperidine, pyridine, 95 °C, 4 h. (ii) Different substituted aromatic phenolic compounds, TFA, CH_2_C_2_, rt, 4 h.

The synthetic route of 4-ferrocenyl-3,4-dihydrocoumatin derivatives (**3a**–**3s**) is shown in Scheme 1. The target compounds were prepared in two steps. First, the commercially available ferrocenecarboxaldehyde (**1**) was reacted with malonic acid in the Knoevenagel reaction to obtain the intermediate (**2**). Then, the intermediate **2** was reacted with substituted aromatic phenolic compounds including phenols and naphthols to afford the target compounds (**3a**–**3s**) in good yields. The structures of compounds **3a**–**3s** are shown in Table 1. All compounds were purified by recrystallisation or column chromatography and characterised by ^1^H NMR, ^13^C NMR, and MS.

**Table 1.** Chemical structures of compounds **3a**–**3s**.

Compd. no.	Structure	Compd. no.	Structure
**3a**		**3k**	
**3b**		**3l**	
**3c**		**3m**	
**3d**		**3n**	
**3e**		**3o**	
**3f**		**3p**	
**3g**		**3q**	
**3h**		**3r**	
**3i**		**3s**	
**3j**			

### Inhibitory activity against LPS-induced NO release

Nitric oxide (NO) is produced by mammalian cells through metabolism of l-arginine and plays an important role in regulating inflammation and immune responses^13^. NO is a key pro-inflammatory mediator and excessive production of NO was proved to be associated with many inflammatory diseases^12^^,^^14^. Thus, it is an established view that compounds inhibiting the production of NO may offer potential opportunity to treat inflammatory diseases. To evaluate the anti-inflammatory activity of these compounds, RAW 264.7 cell was used as a model cell. RAW 264.7 cells were pre-incubated with all target compounds (20 µM) for 1 h and treated with LPS (1.0 µg/mL) for 24 h. The cell conditioned medium was collected and the NO content in the media was detected by the Griess reagent assay.

**Figure 2.**
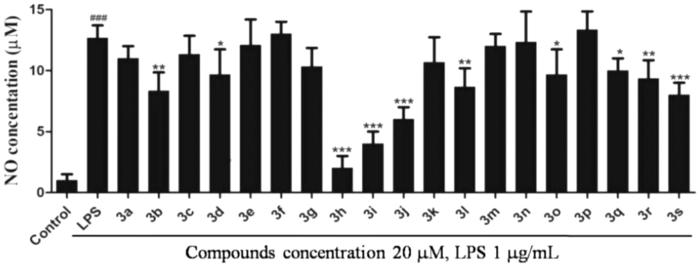
Inhibition of NO production by compounds **3a**–**3s**. RAW 264.7 cells were pretreated with compounds **3a**–**3s** (20 μM) for 1 h and treated with LPS (1 μg/mL) for 24 h. ^###^*p*<.001 compared with control group; *p<.05, **p<.01, and ***p<.001 compared with LPS treated group.

The screening results indicated that most of the target compounds could significantly reduce the LPS-induced NO secretion at a dosage of 20 µM (Figure 2). The inhibitory rates (IRs) of all the target compounds were higher than 85%. In addition, the IR of compounds **3b**, **3d**, **3h**, **3i**, **3j**, **3l**, **3o**, **3q**, **3r**, and **3s** were higher than 90%. Among them, compounds **3h**, **3i**, and **3j** showed stronger inhibition of NO compared to the positive compound Celecoxib. It is obvious that compound **3h** exhibited the most potent inhibitory activity against NO production (IR > 95%) compared with Celecoxib. According to the results, the structure–activity relationship (SAR) can be easily summarised that introduction of coplanar groups like naphthalenyl (**3h** and **3i**) and benzodioxol (**3j**) bridging to the dihydrocoumarin ring is beneficial to the NO inhibitory activity.

### Toxicity assessment

**Figure 3.**
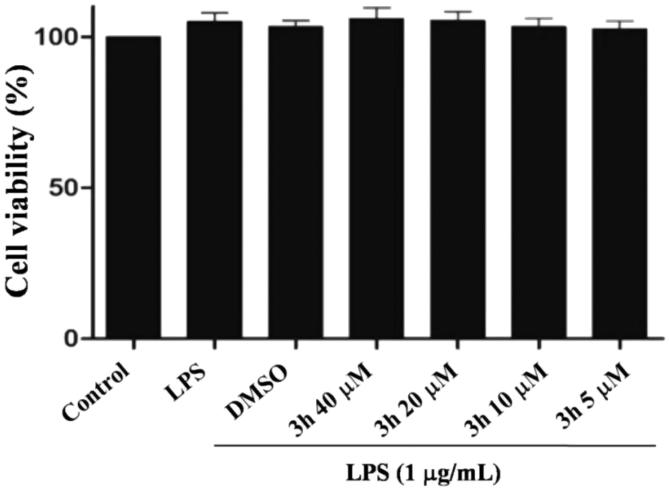
The cytotoxic evaluation of compound **3h** against Raw 264.7 cells. RAW 264.7 cells were pretreated with DMSO or different concentrations of compound **3h** for 1 h and treated with LPS (1 μg/mL) for 24 h.

Preliminary screening results showed that most of the target compounds had good anti-inflammatory activity. To confirm the possibility for further research, the cytotoxicity of compound **3h** was evaluated by the MTT assay in RAW264.7 cells. As shown in Figure 3, compound **3h** demonstrated low toxicity even at a high concentration of 40 µM, indicating that compound **3h** is worthy of further evaluation.

### Inhibition of the cytokines production by compound 3h

**Figure 4.**
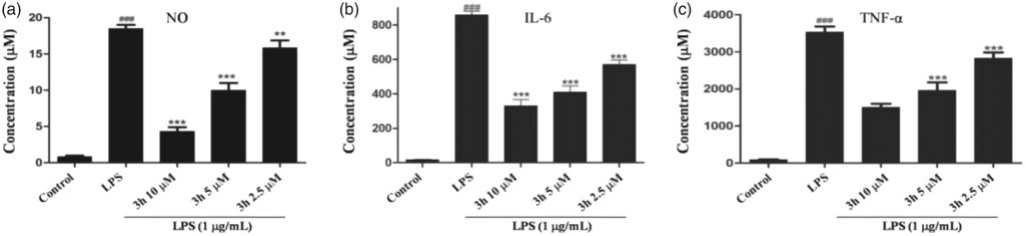
Inhibition of the cytokines production by compound **3h.** RAW 264.7 cells were pretreated with compound **3h** at concentration of 10, 5, and 2.5 μM for 1 h and treated with LPS (1 μg/mL) for 24 h. (A) NO production was measured using nitrite and nitrate assay. (B and C) TNF-α and IL-6 levels in the medium were determined with an ELISA kit. ^###^*p*<.001 compared with control group; **p<.01 and ***p<.001 compared with LPS treated group.

The pro-inflammatory cytokines including IL-6 and TNF-α are involved in the pathogenesis of RA. It has been reported that TNF-α and IL-6 participate in synovial proliferation and induced cartilage destruction^15^. TNF-α was particularly proven to be a key pathogenic inflammatory cytokine of RA^16^. The autocrine production of TNF-α by RA bone erosion could promote the expression of other inflammatory mediators and induce immune cell infiltration to aggravate RA disease^17^. In this study, RAW 264.7 cells were pretreated with compound **3h** at a series of concentrations (2.5, 5, and 10 μM) for 1 h and then with LPS (1 µg/mL) for 24 h. The levels of TNF-α and IL-6 in cell culture were evaluated by ELISA. As shown in Figure 4, compound **3h** significantly reduced NO, IL-6, and TNF-α levels in cell culture in a concentration-dependent manner. Based on the results above, **3h** was an effective anti-inflammatory compound. Thus, compound **3h** was selected as the title compound for further mechanism exploration.

### Mechanism exploration of anti-inflammatory activity

#### Compound 3h suppresses LPS-induced inflammatory response

**Figure 5.**
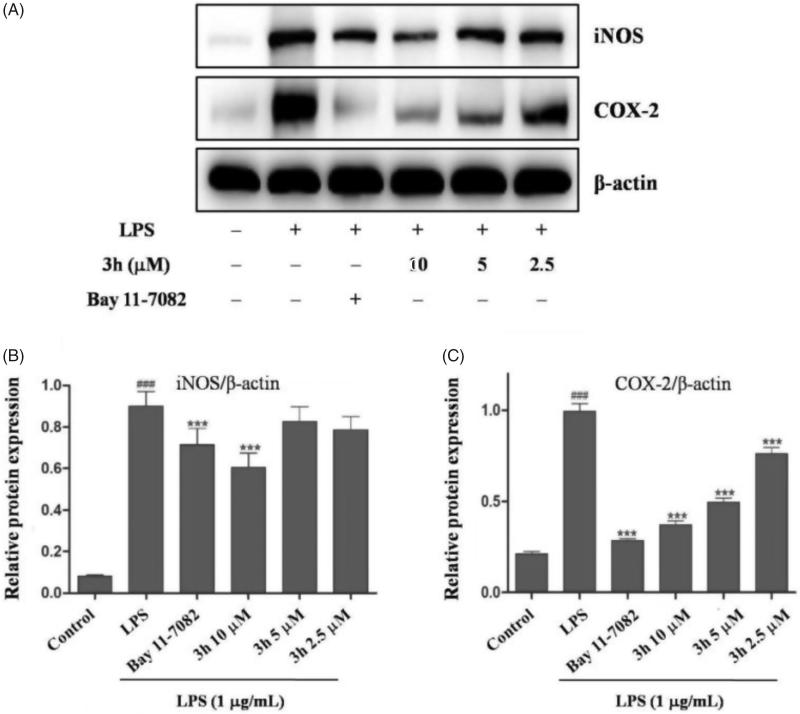
Compound **3h** inhibited the LPS-induced expression of iNOS and COX-2. RAW 264.7 cells were pretreated with compound **3h** at concentration of 10, 5, and 2.5 μM for 1 h and treated with LPS (1 μg/mL) for 24 h. (A) Protein expression of iNOS and COX-2 was detected by Western blotting analysis. (B and C) Semi-quantitative statistical graph of iNOS and COX-2 protein expressions in various groups. Bay 11-7082 was positive control and β-actin was used as loading control. ^###^*p*<.001 compared with control group; ***p<.001 compared with LPS treated group.

NO is synthesised by nitric oxide synthase (NOS). NOS isoforms include neuronal nitric oxide synthase (nNOS), inducible nitric oxide synthase (iNOS), and endothelial nitric oxide synthase (eNOS)^18^. Many inflammatory conditions are associated with production of large amounts of NO and iNOS. The findings have confirmed that iNOS inhibitors exert beneficial effects on osteoarthritis^19^. Moreover, the cyclooxygenase-2 (COX-2) has been well-studied to play an important role in RA which is induced in inflammatory situations and highly expressed in synovial tissues of RA patients^20^^,^^21^. Thus, the inhibitory effects of compound **3h** on LPS-mediated expressions of iNOS and COX-2 were analysed by Western blotting. As shown in Figure 5, the LPS (1 µg/mL) stimulation for 24 h could markedly augmented iNOS and COX-2 expression. Additionally, compound **3h** could dose-dependently suppress LPS-induced iNOS and COX-2 expression. These results further demonstrated that compound **3h** could prevent LPS-induced inflammatory response in macrophages.

#### Inhibition of LPS-induced NF-κB signalling pathway activation

Nuclear factor-κB (NF-κB) is a principal transcription factor for amplifying expression of many cytokines that are involved in the pathogenesis of inflammatory diseases^22^. In patients with RA, activation of NF-κB signalling pathway results in production of inflammatory cytokines including TNF-α and IL-6^23^. The NF-κB family includes NF-κB1, NF-κB2, P65, RelB, and c-Rel. Among NF-κB family, P65 plays the most important role in the development of inflammation^24^. Many inflammation cytokines such as LPS, TNF-α, and IL-1β can stimulate the activation of IκB kinase (IKK). The activated IKK then phosphorylates IκB which regulates the nuclear translocation of NF-κB^25^.

**Figure 6.**
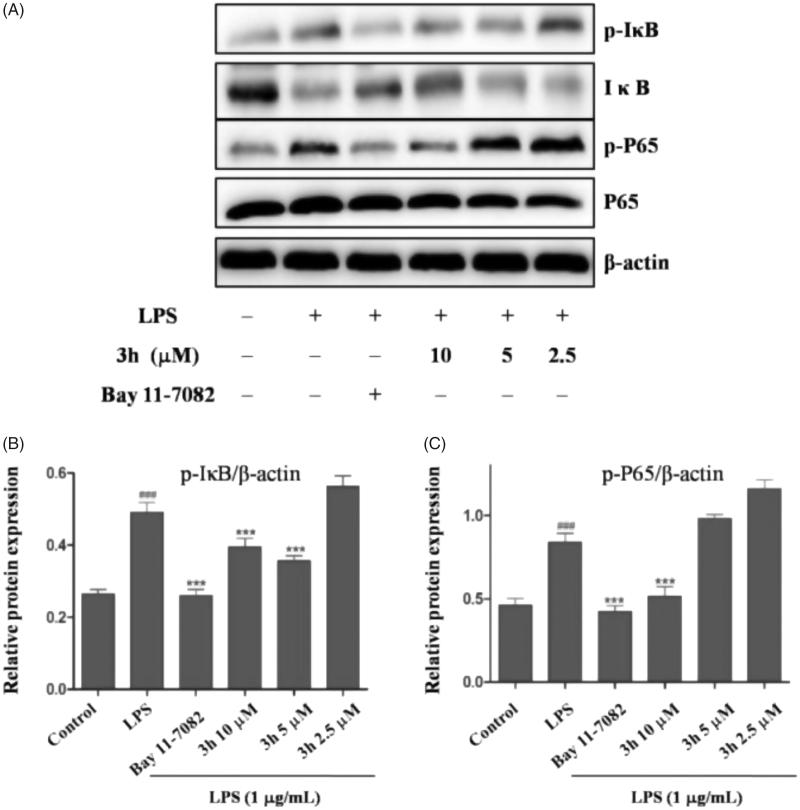
Compound **3h** inhibited LPS-induced activation of NF-κB signalling pathway in RAW 264.7 cells. RAW 264.7 cells were pretreated with compound **3h** at concentration of 10, 5, and 2.5 μM for 1 h and treated with LPS (1 μg/mL) for 24 h. (A) Proteins expression were detected by Western blotting analysis. (B and C) Semi-quantitative statistical graph of p-IκB, IκB and p-P65 protein expressions in various groups. Bay 11-7082 was positive control and β-actin was used as loading control. ^###^*p*<.001 compared with control group; ***p<.001 compared with LPS treated group.

To explore the anti-inflammatory mechanism of compound **3h**, we examined the effect of compound **3h** on the markers of the NF-κB pathway. The Western blotting analyses demonstrated that the expression levels of p-IκB and p-P65 were suppressed in compound **3h** group compared with untreated group, indicating that compound **3h** could decrease the IκB phosphorylation level and degradation (Figure 6). Meanwhile, it could also inhibit NF-κB P65 translocate into the nucleus.

#### Compound 3h inhibits LPS-induced ERK signalling activation

**Figure 7.**
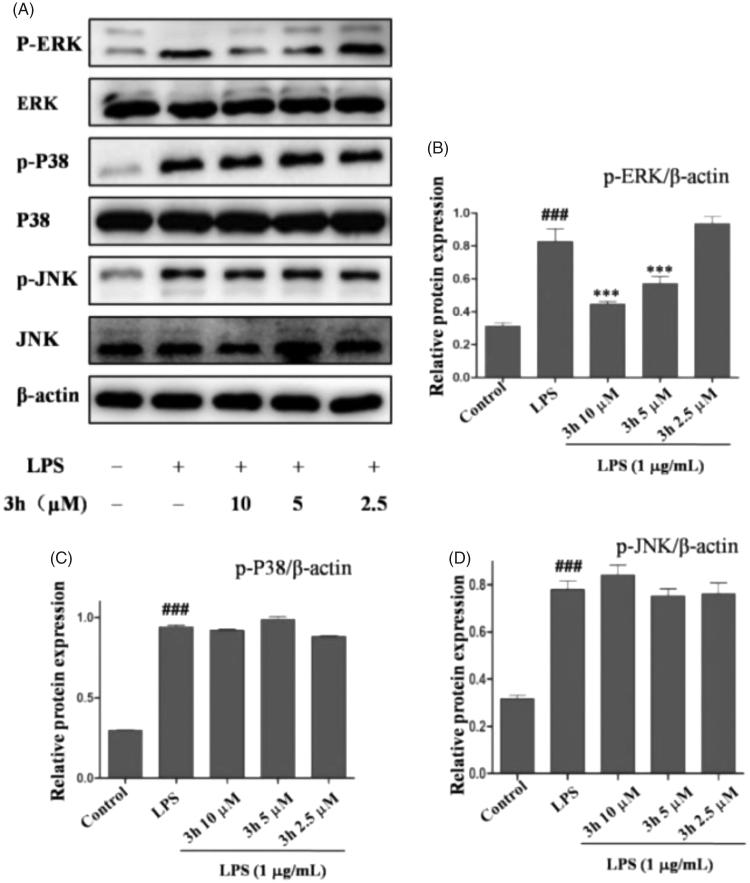
Compound **3h** inhibited LPS-induced MAPKs signalling pathway. RAW 264.7 cells were pretreated with compound **3h** at concentration of 10, 5, and 2.5 μM for 1 h and treated with LPS (1 μg/mL) for 24 h. (A) Proteins expression were detected by Western blotting analysis. (B, C and D) Semi-quantitative statistical graph of p-ERK, p-P38 and p-JNK protein expressions in various groups. ^###^*p*<.001 compared with control group; ***p<.001 compared with LPS treated group.

Mitogen-activated protein kinases (MAPKs) are the key signalling pathway that plays an important role in RA^26^. To further investigate the anti-inflammatory mechanism of compound **3h** on CFA-induced arthritis, the inflammation-related signalling pathway of MAPK was studied. As shown in Figure 7, the expression levels of p-ERK, p-P38, and p-JNK were upregulated in rats after exposure to CFA. Treatment with compound **3h** effectively downregulated the protein levels of p-ERK in a dose-dependent manner. The three major kinase cascades in MAPK signalling pathway include extracellular signal regulated kinase (ERK), C-Jun-terminal kinase (JNK), and P38 MARK. In the current study, compound **3h** treatment caused significant inhibition of the phosphorylation of ERK. The results indicated that compound **3h** exert its anti-inflammatory action by inhibiting the activation of ERK/MAPK pathway.

#### In vivo anti-inflammatory activity of compound 3h

**Figure 8.**
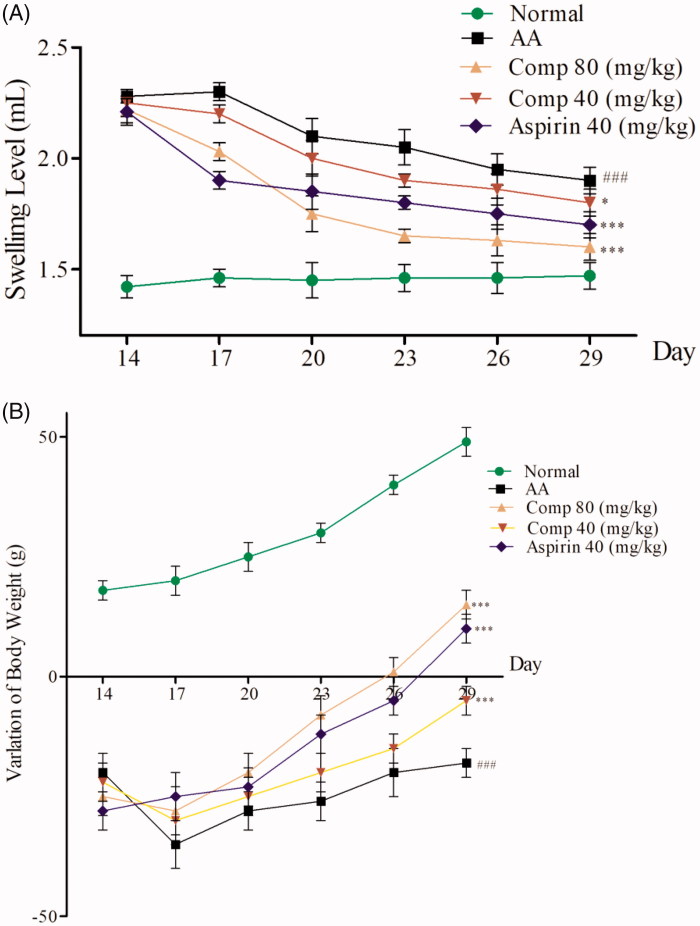
(A) Effect of compound 3h on paw swelling in rats with adjuvant arthritis (AA); (B) Effect of compound 3h on weight in rats with adjuvant arthritis. Data are the mean ± SD, *n* = 5 per group. Aspirin was the positive control: ^###^*p*<.01 compared with control; ∗∗∗*p*<.001, ∗*p*<.05 vs. AA group.

The CFA-induced arthritis (AIA) is a widely used model to study RA^27^. In this study, we established the rat AIA model by intradermal injection of CFA into the right hind paw^14^. We observed that 80–90% of rats exhibited obvious hind paw swelling and body weight loss within 14–28 days after CFA injection. AIA rats were given intragastric administration of compound **3h** once a day from day 14 to 28, aspirin was given intragastrically as the positive control. The results are shown in Figure 8.

**Figure 9.**
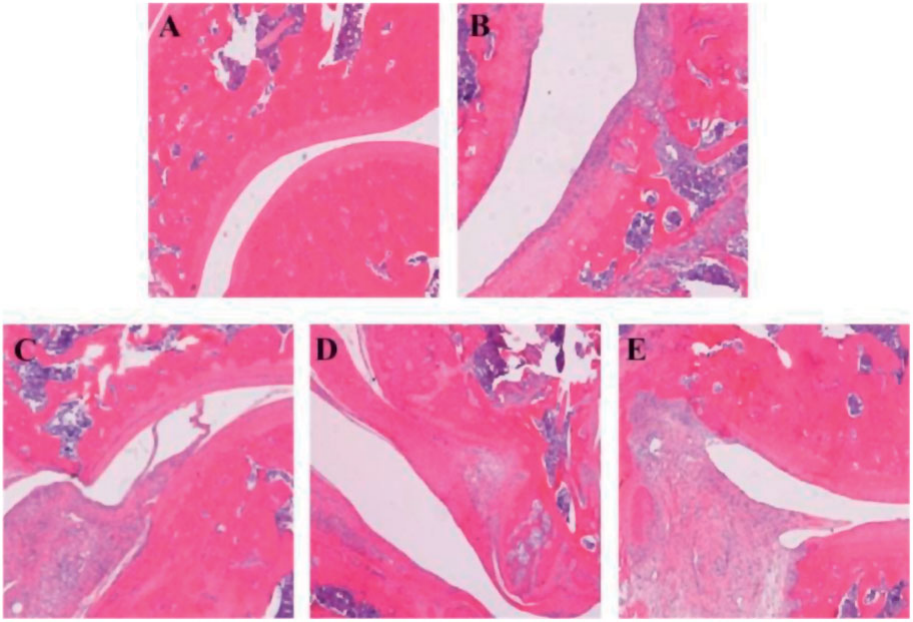
Typical histopathological images of knee joints illustrated the anti-inflammatory effects of title compound **3h** on AIA rats: (A) normal; (B) AIA; (C) aspirin, 40 mg/kg; (D) compound **3h**, 40 mg/kg; (E) compound **3h**, 80 mg/kg. Aspirin was the positive control.

Histopathological analysis of knee joints was conducted at the end of the experiment to evaluate the level of inflammation and changes of the tissue. Representative histological photographs of the tissue sections are presented in Figure 9. No inflammation was seen in normal rats (Figure 9(A)). AIA rats exhibited extensive inflammatory cells infiltration, synovial hyperplasia, and cartilage destruction (Figure 9(B)). Administration of compound **3h** at dose of 40 mg/kg significantly reduced the infiltration of inflammatory cells and improved synovial hyperplasia and cartilage destruction (Figure 9(D)). Escalating the dose of compound **3h** (80 mg/kg) resulted in a better therapeutic effect on AIA rats (Figure 9(E)). Taken together, the results of histopathological evaluation suggested that compound **3h** was able to attenuate inflammation of AIA rats.

## Conclusions

In summary, nineteen 4-ferrocenyl-3,4-dihydrocoumarin derivatives were designed and synthesised to discover novel agents with anti-inflammatory activity. The preliminary screening results indicated that most target compounds exhibited significant NO inhibitory activity with low toxicity against RAW 264.7 cells. Based on the SAR, the introduction of coplanar groups like naphthalenyl and benzodioxol bridging to the dihydrocoumarin ring is beneficial to the NO inhibitory activity. Furthermore, the compound **3h** was found to be able to inhibit the secretion of IL-6, NO, and TNF-α in a dose-dependent manner. The preliminary mechanism investigation indicated that compound **3h** could significantly suppress LPS-induced expressions of iNOS, COX-2, IL-6, TNF-α, and NO via inactivating the NF-κB and ERK/MAPK signalling pathways. The further *in vivo* anti-inflammatory study revealed that compound **3h** effectively relieved the histological changes in knee joints of AIA rats.

## Supplementary Material

Supplemental Material
