# Supplementary figures and images for: Synthesis and in vitro and in vivo anti-inflammatory activity of novel 4-ferrocenylchroman-2-one derivatives
Source: J Enzyme Inhib Med Chem. 2019 Sep 17;34(1):1678–89. doi: 10.1080/14756366.2019.1664499 (PMC6758610; doi:10.1080/14756366.2019.1664499)

## Supplementary information

### $^1\text{H}$ , $^{13}\text{C}$ NMR spectra for target compounds

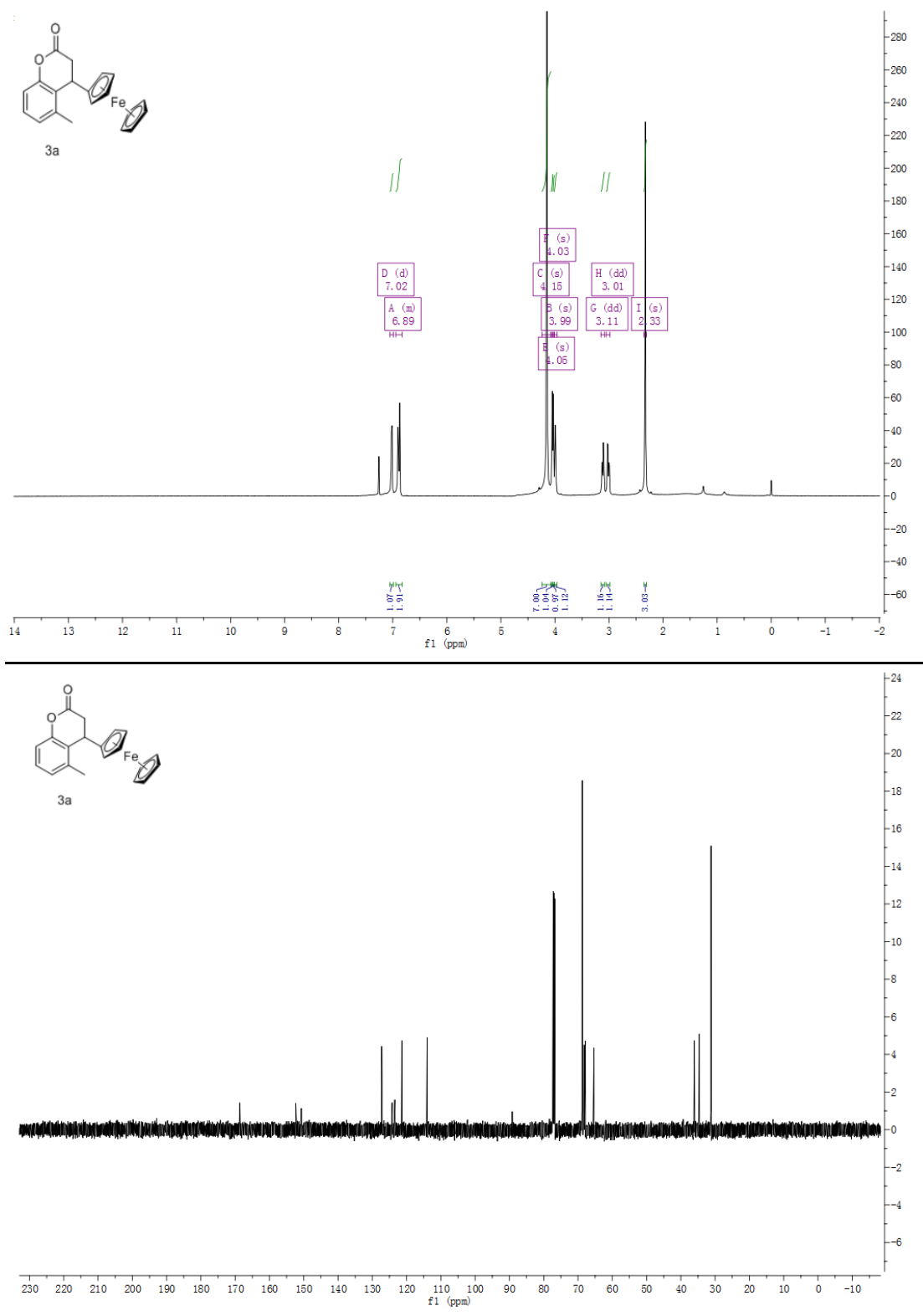

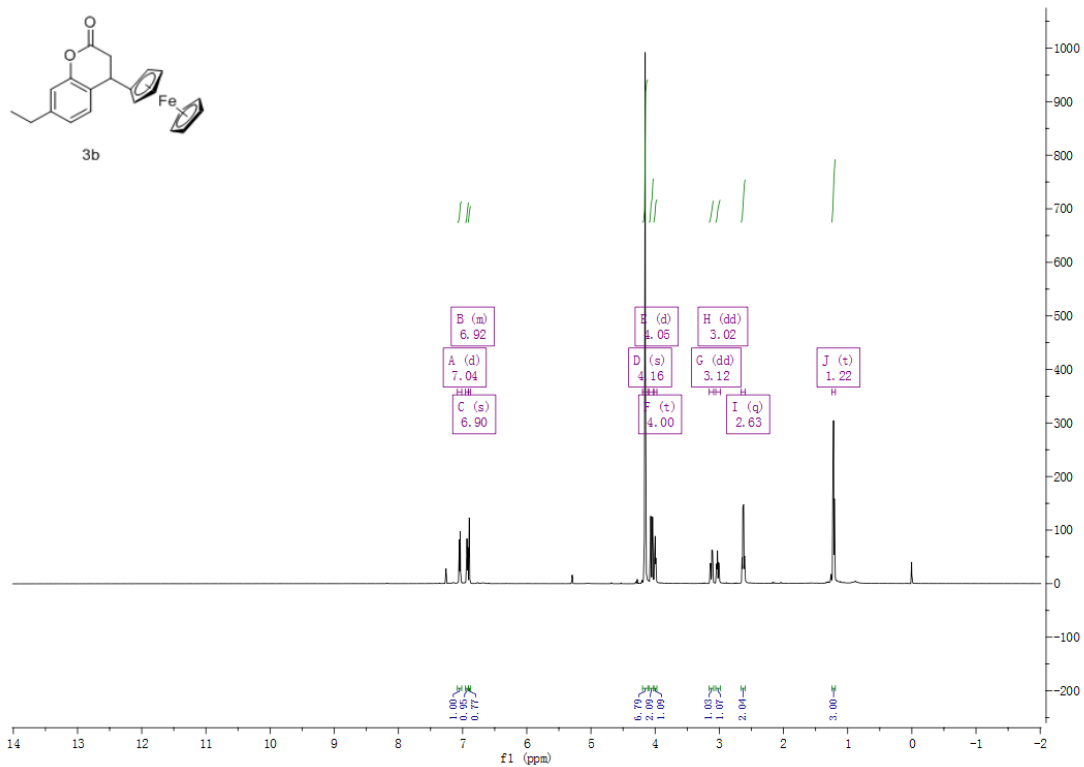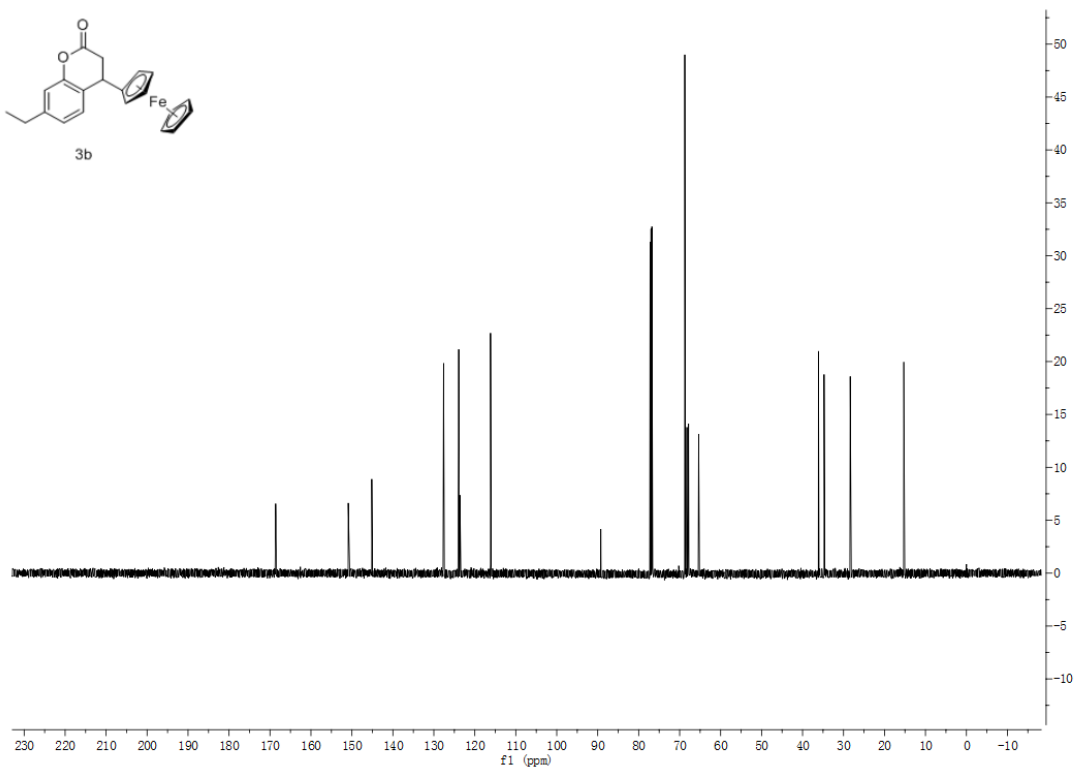

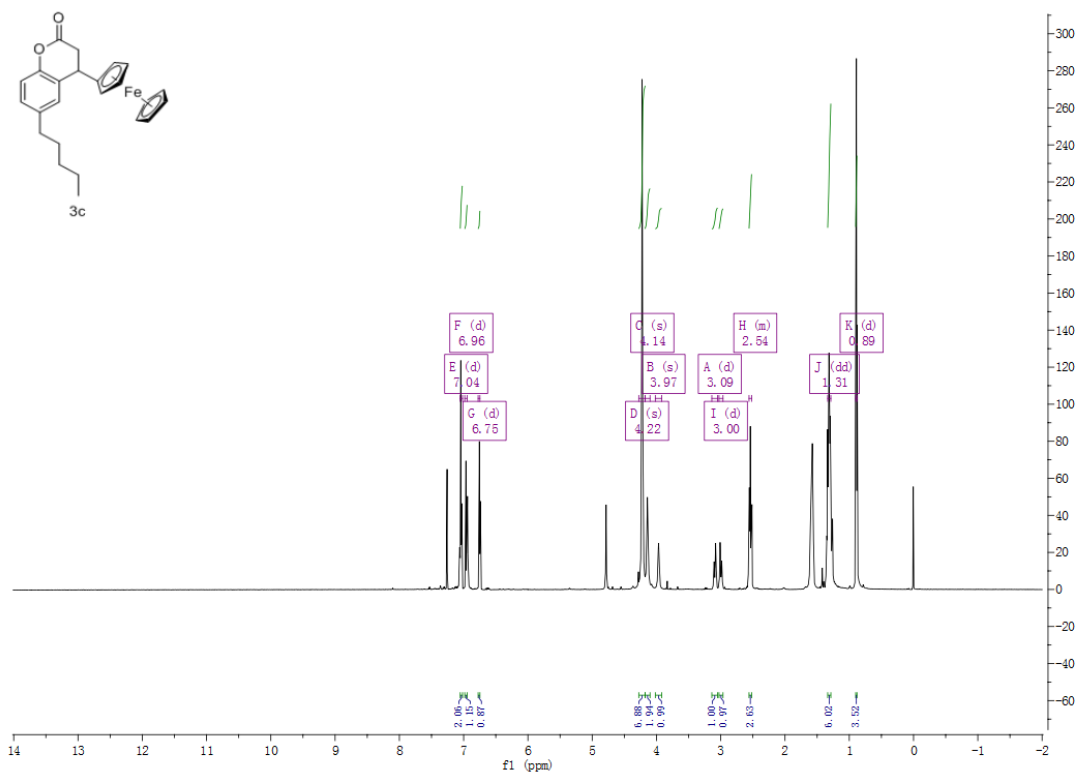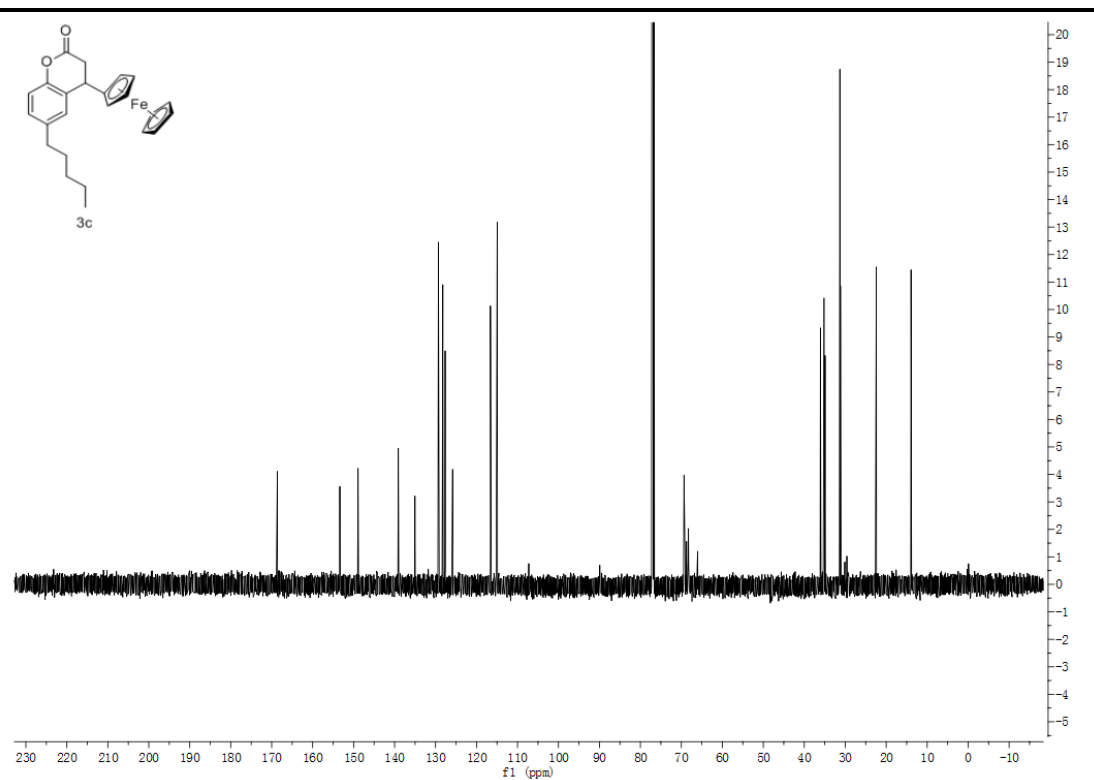

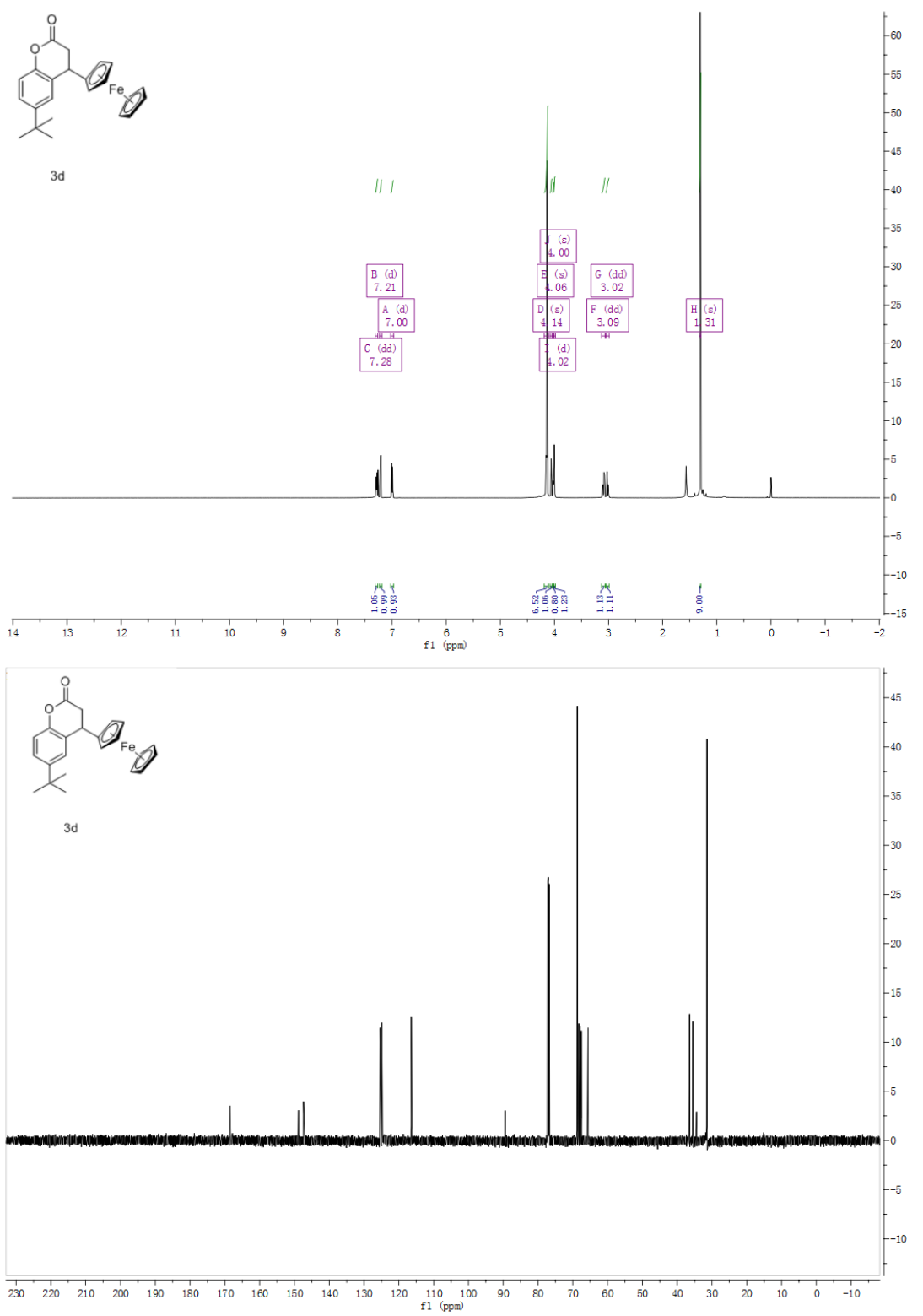

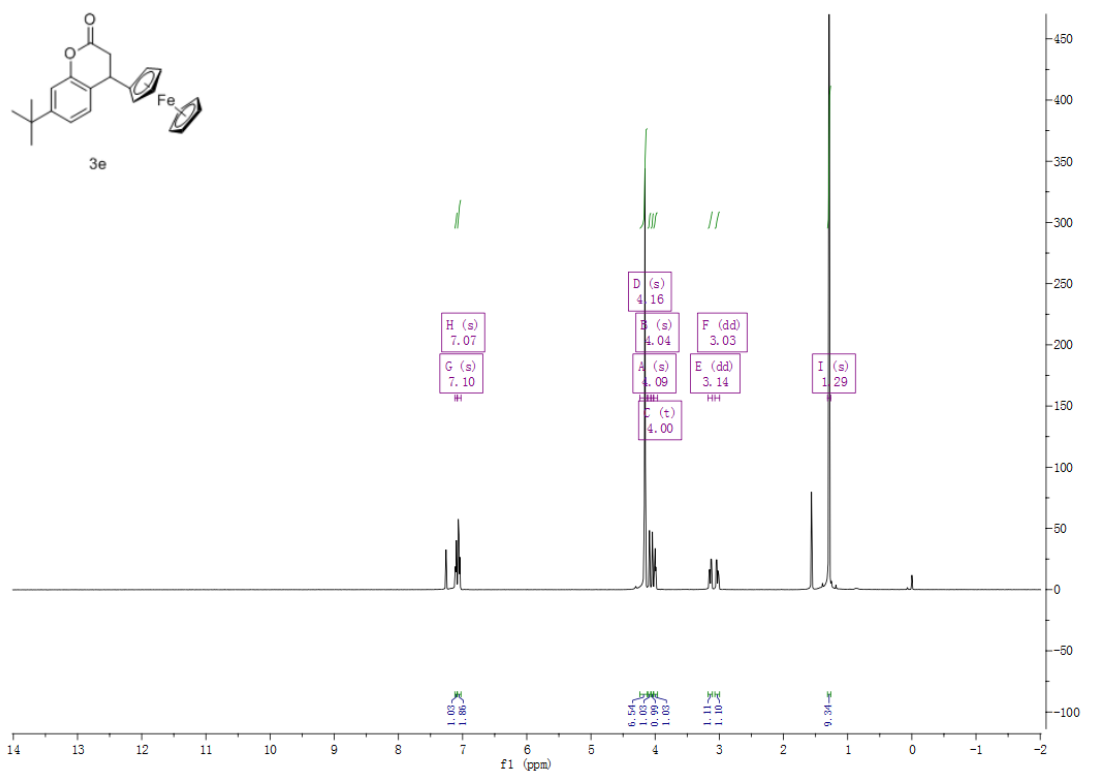

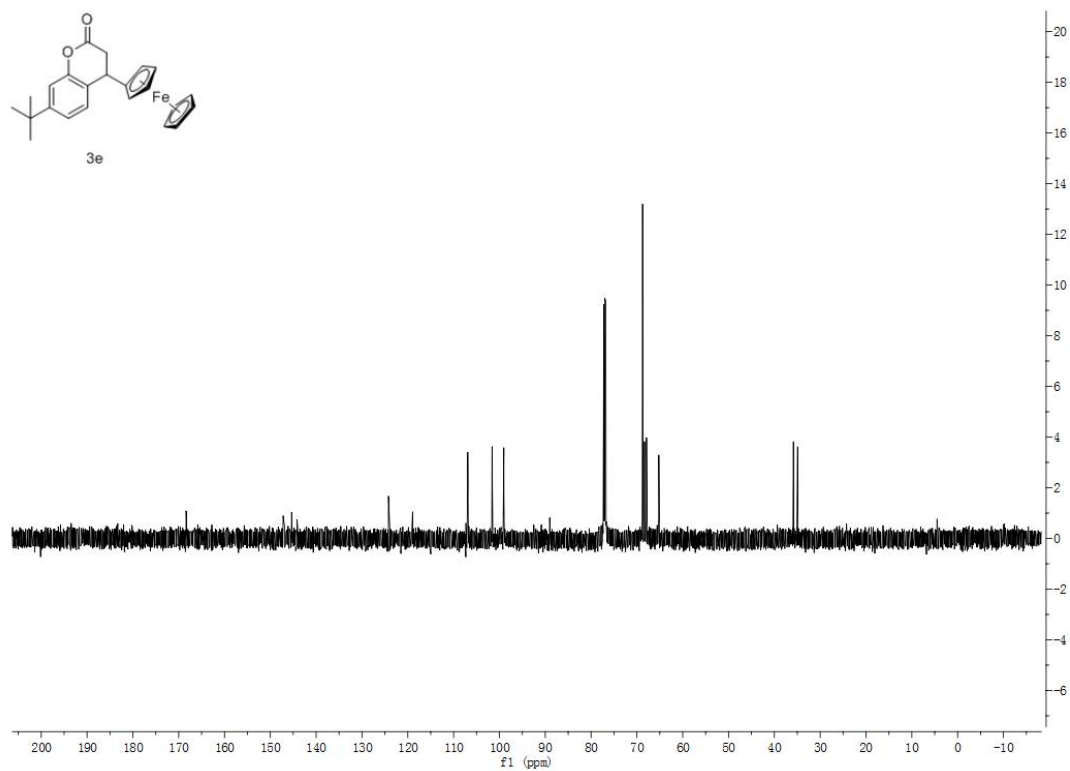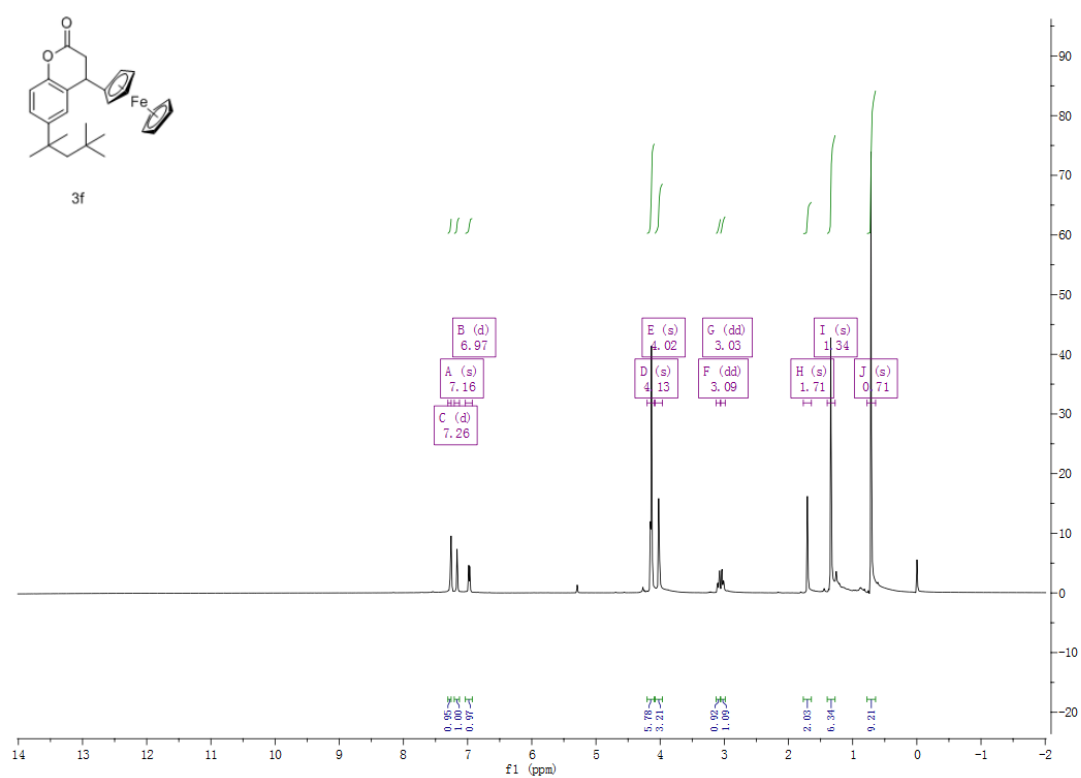

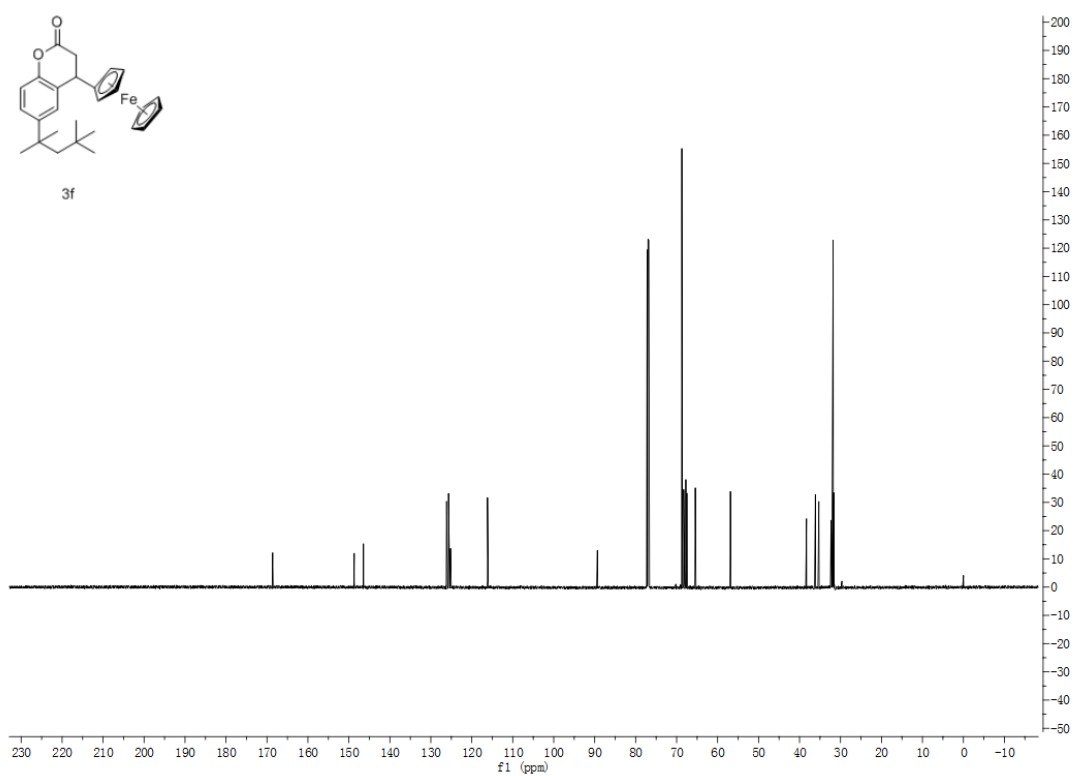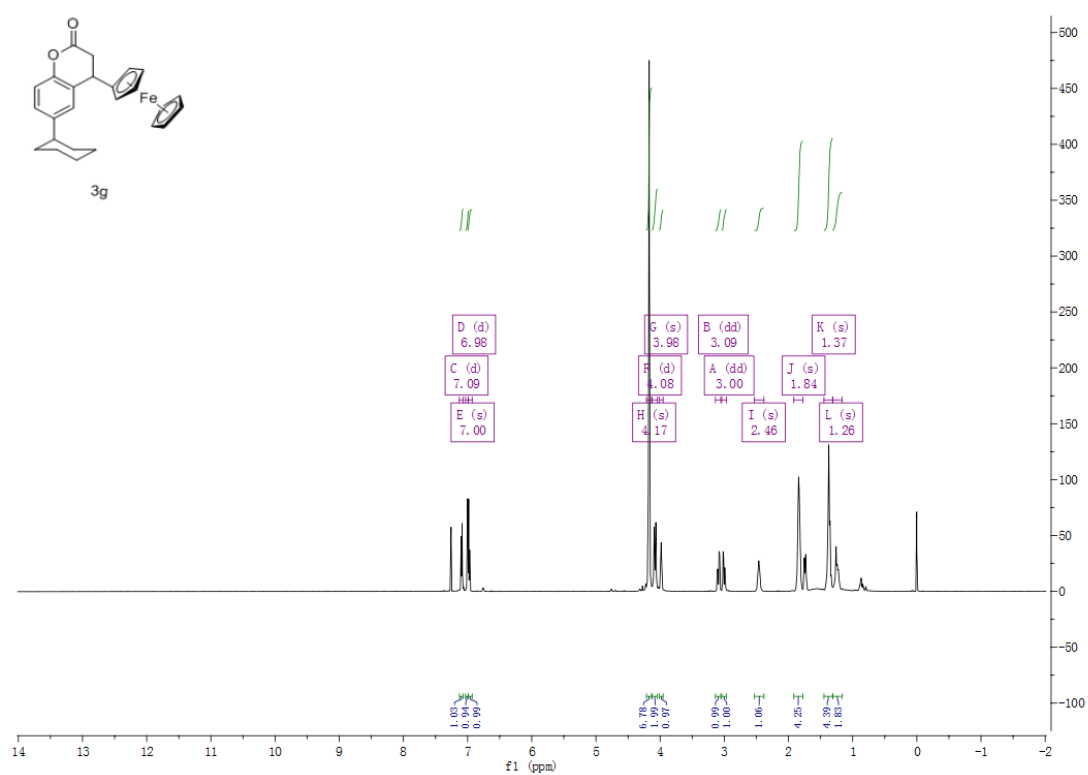

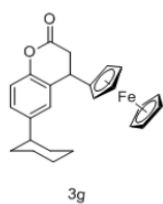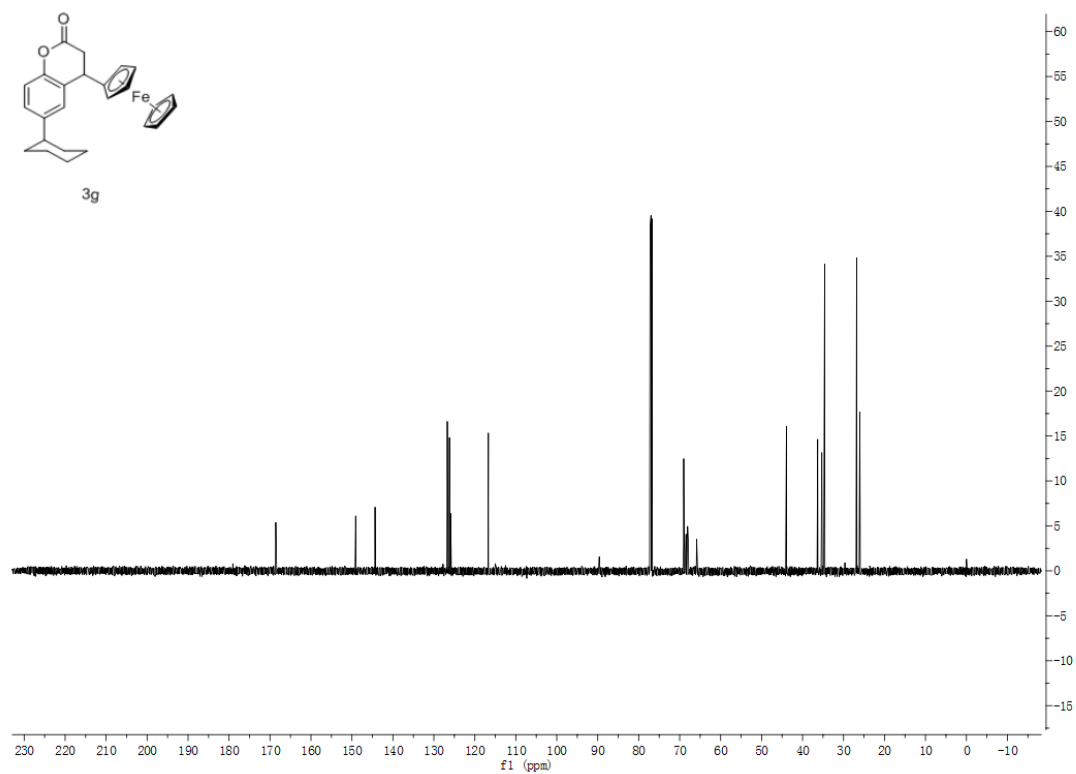

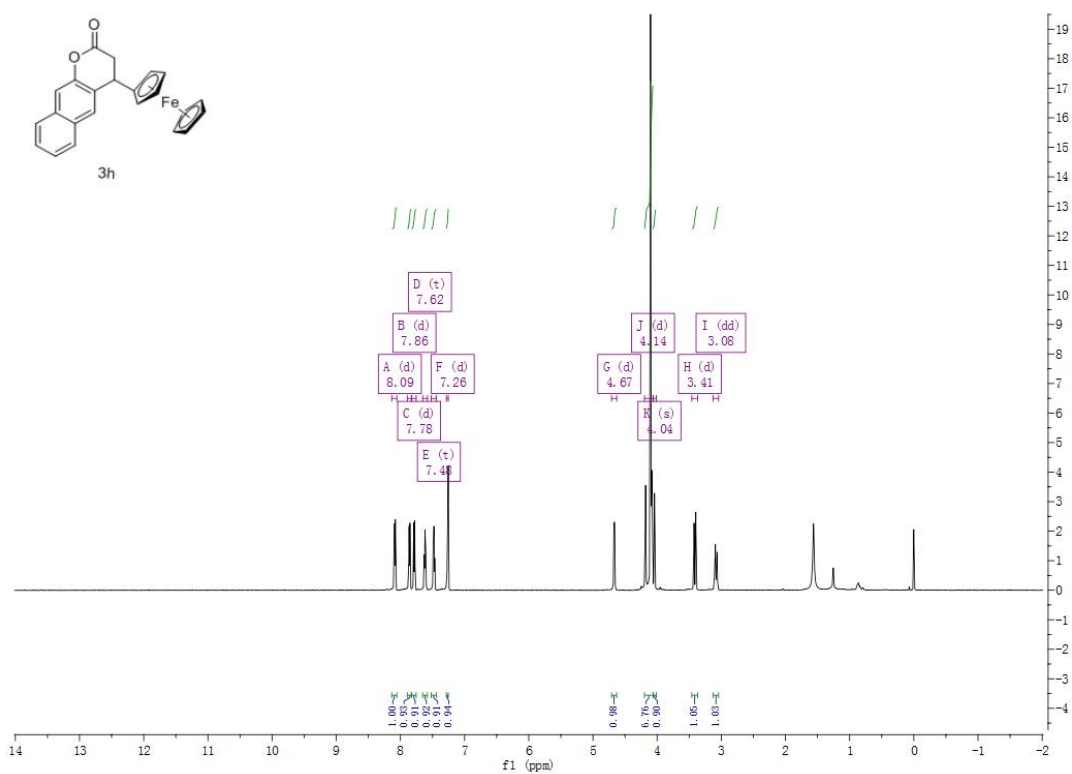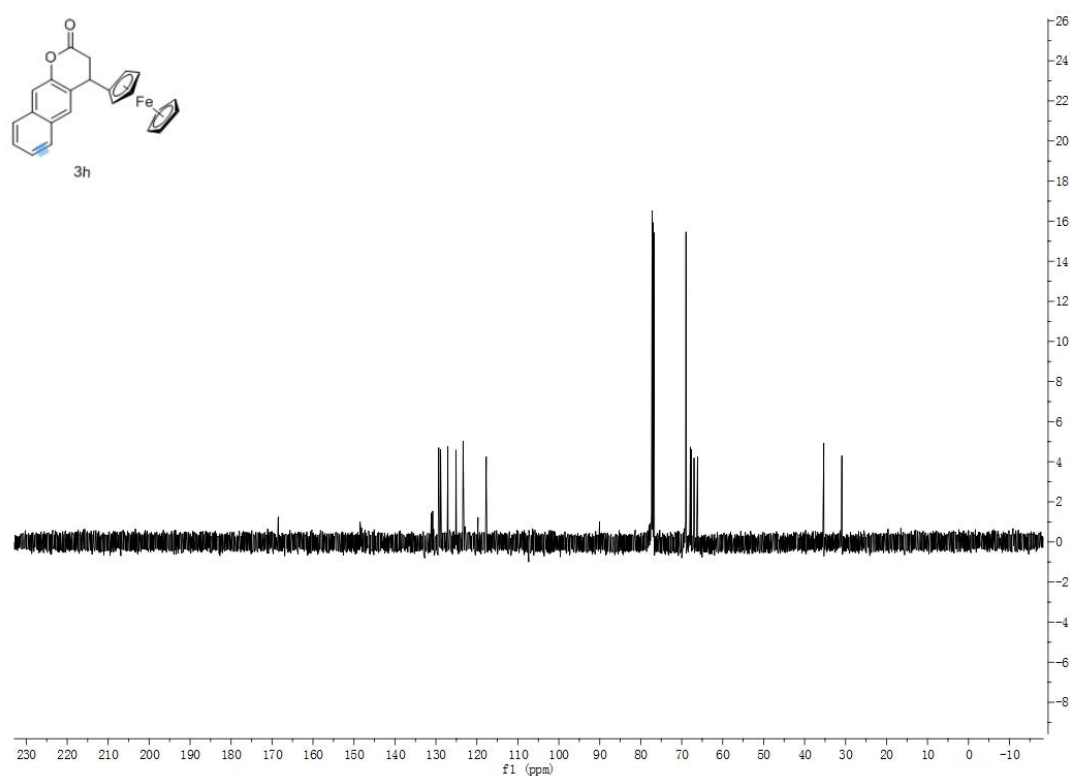

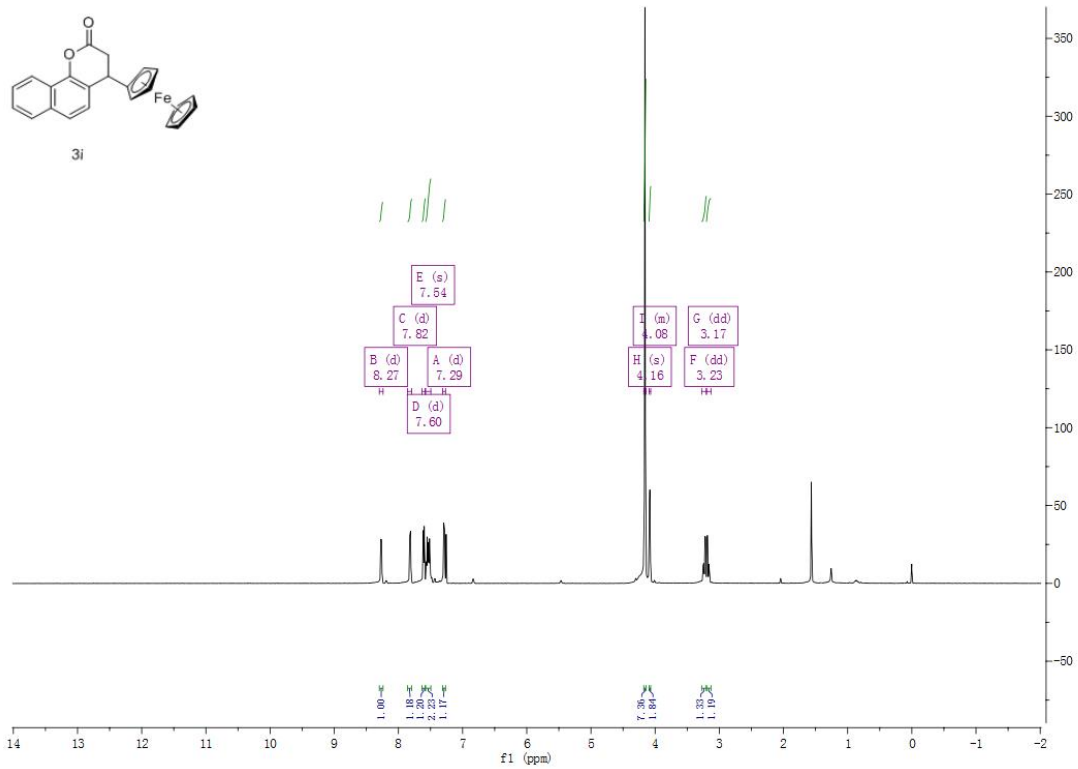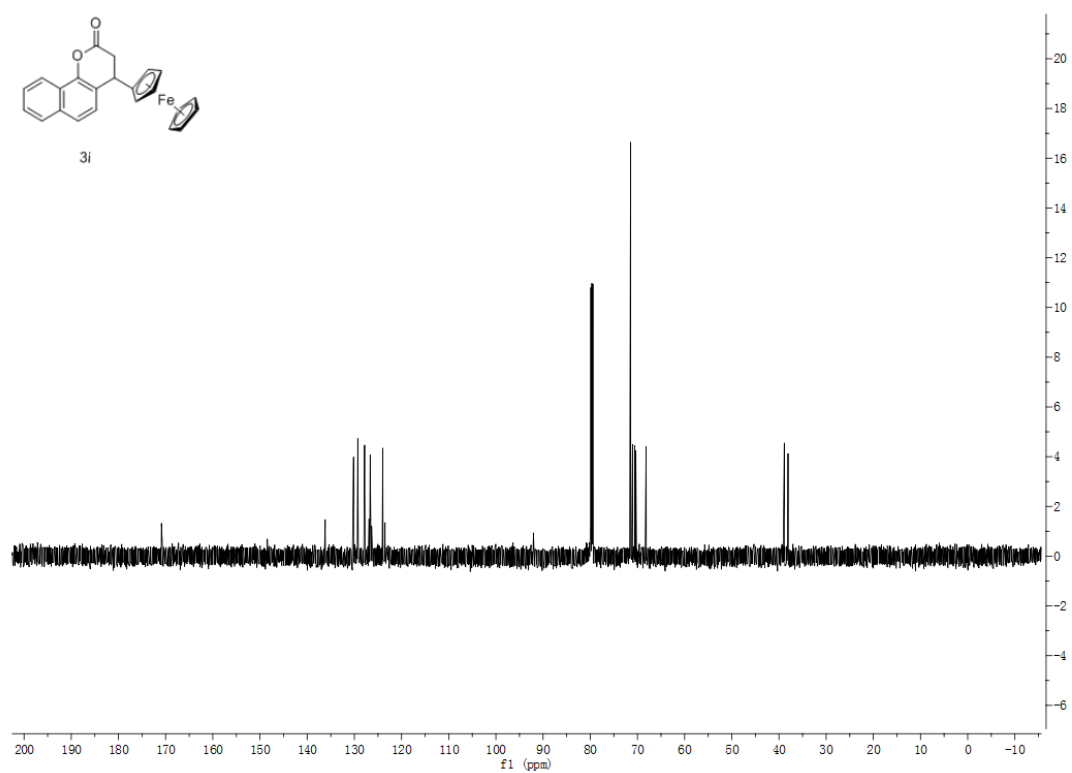

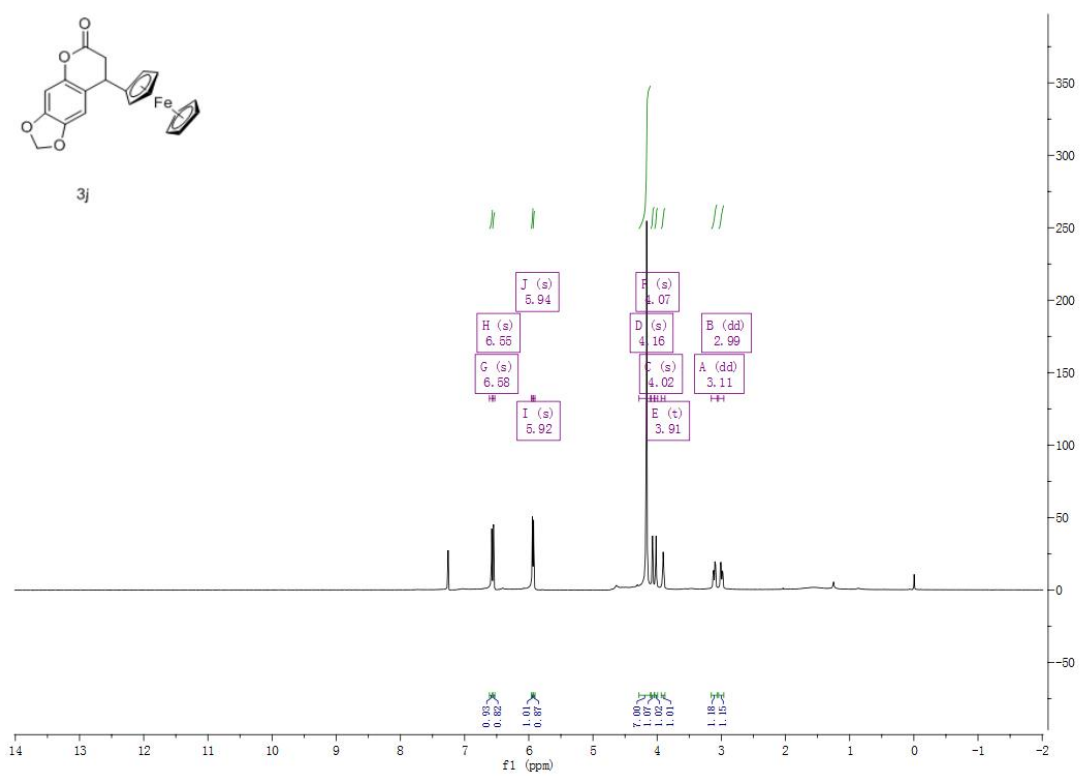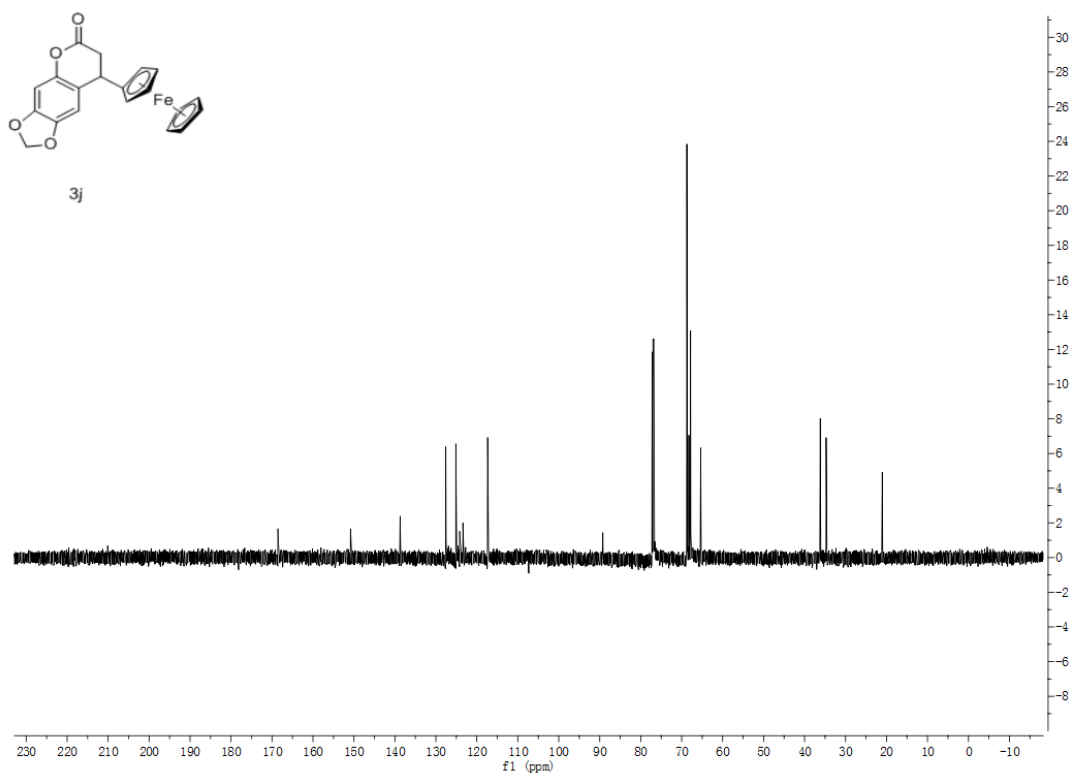

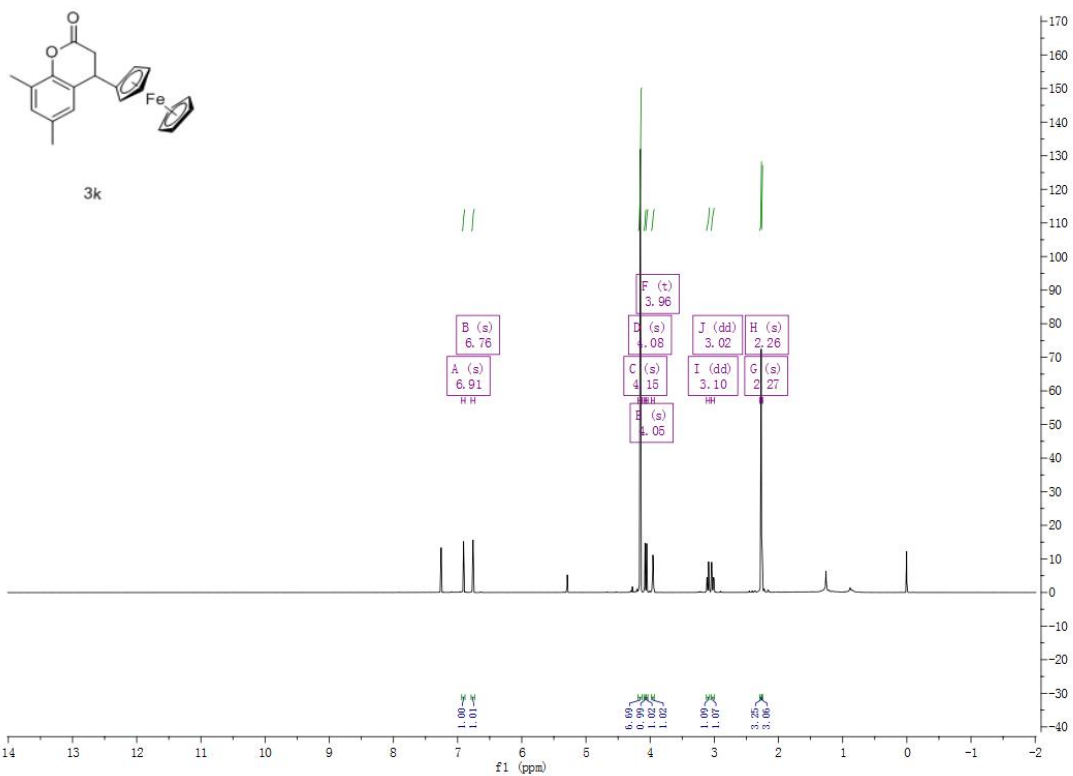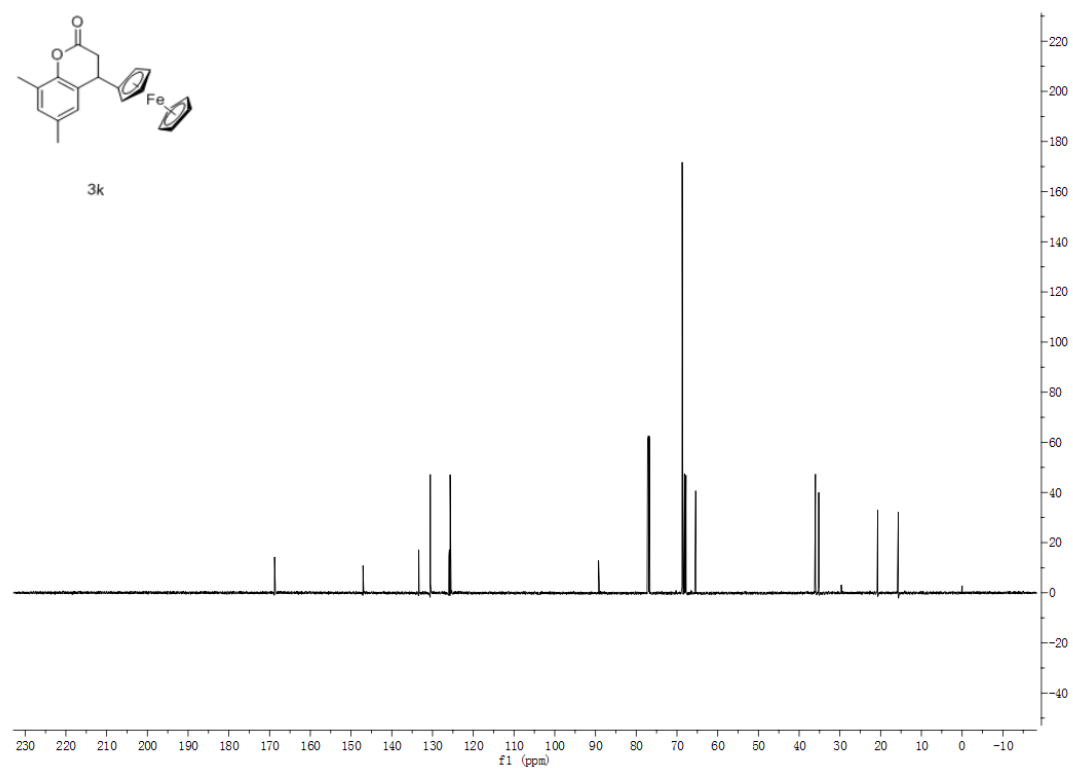

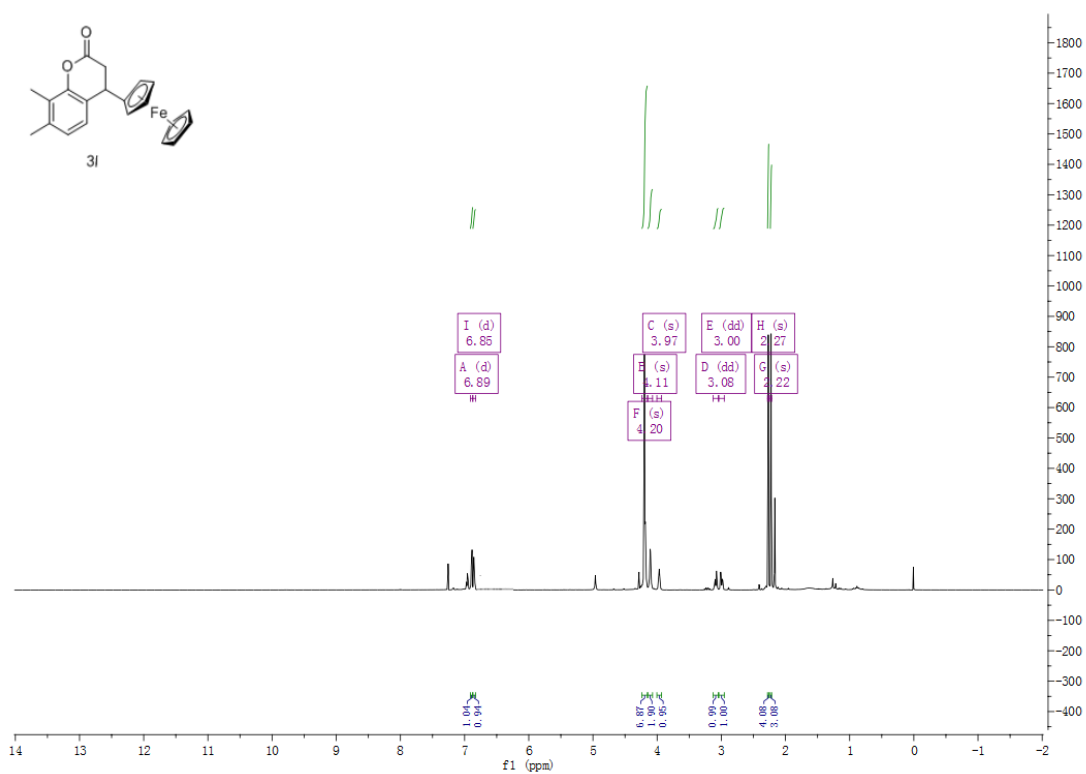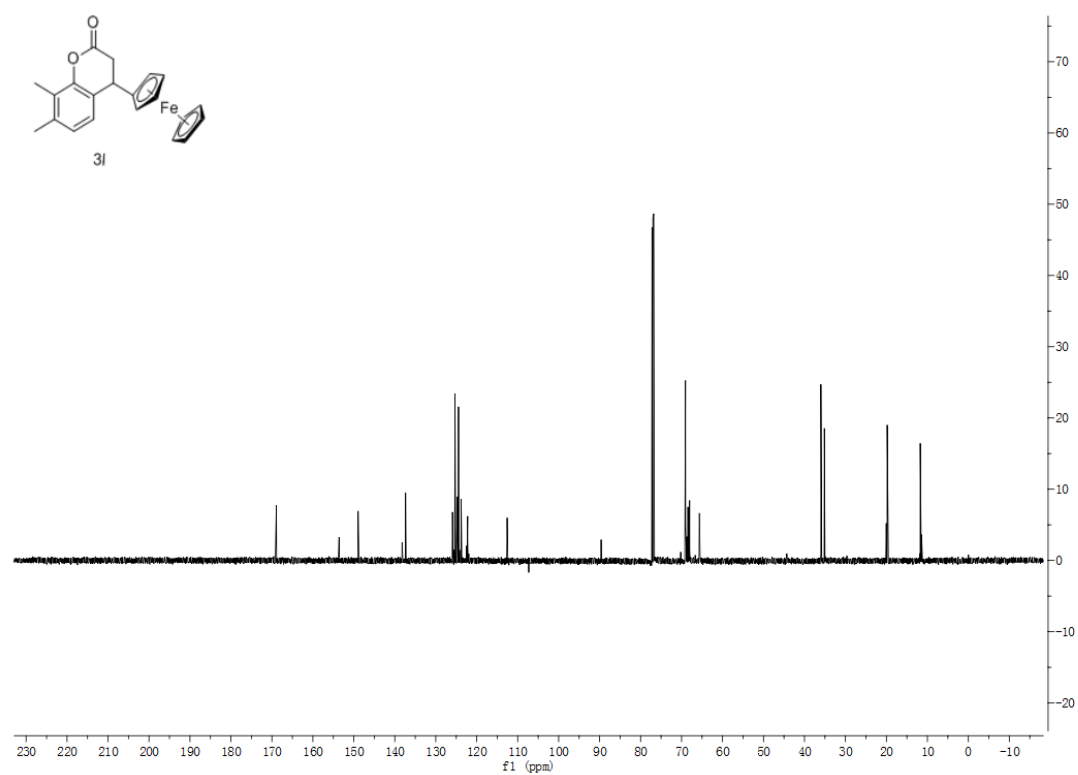

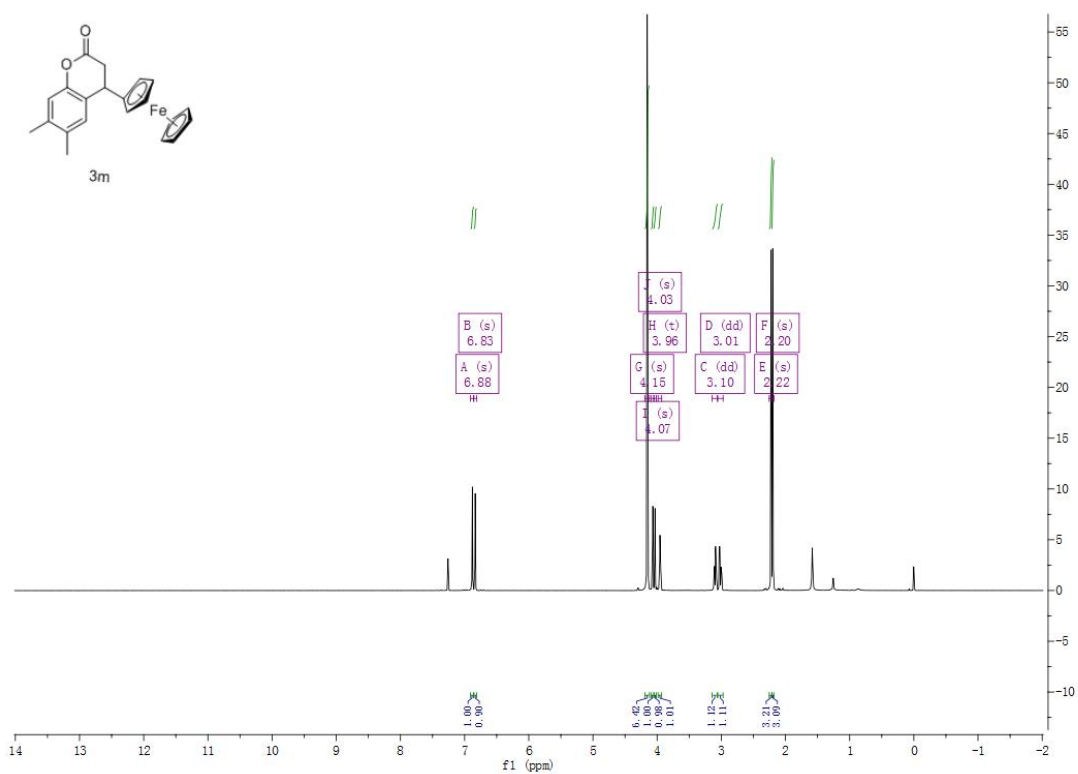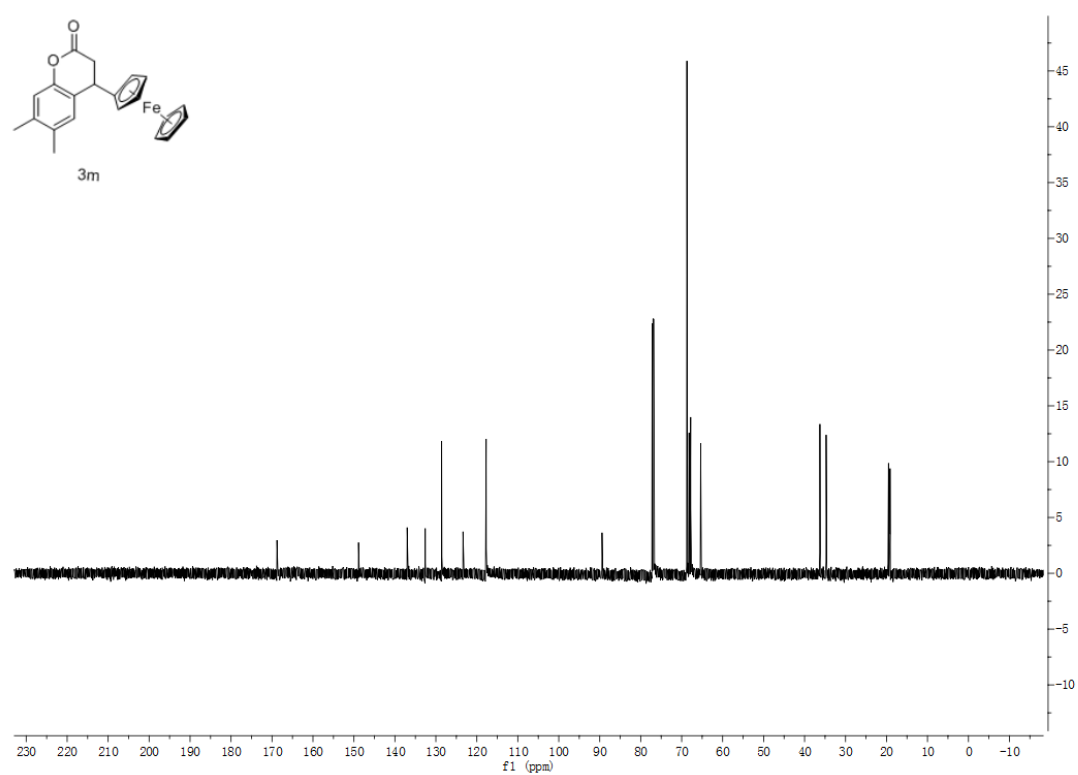

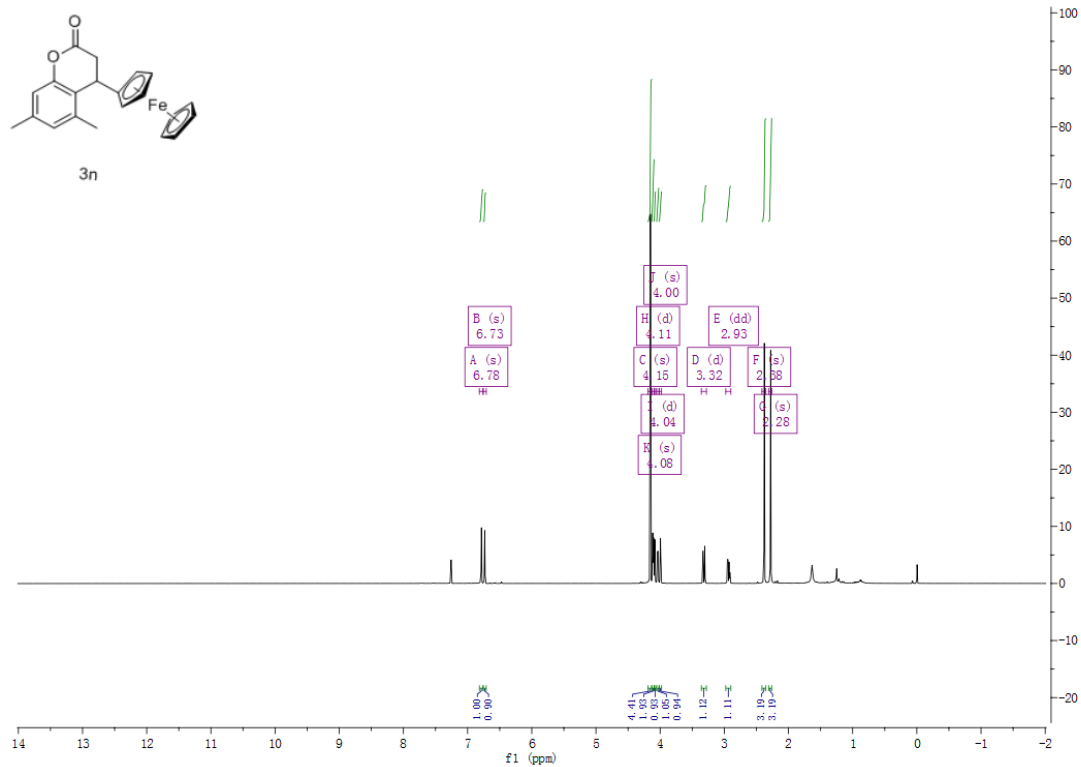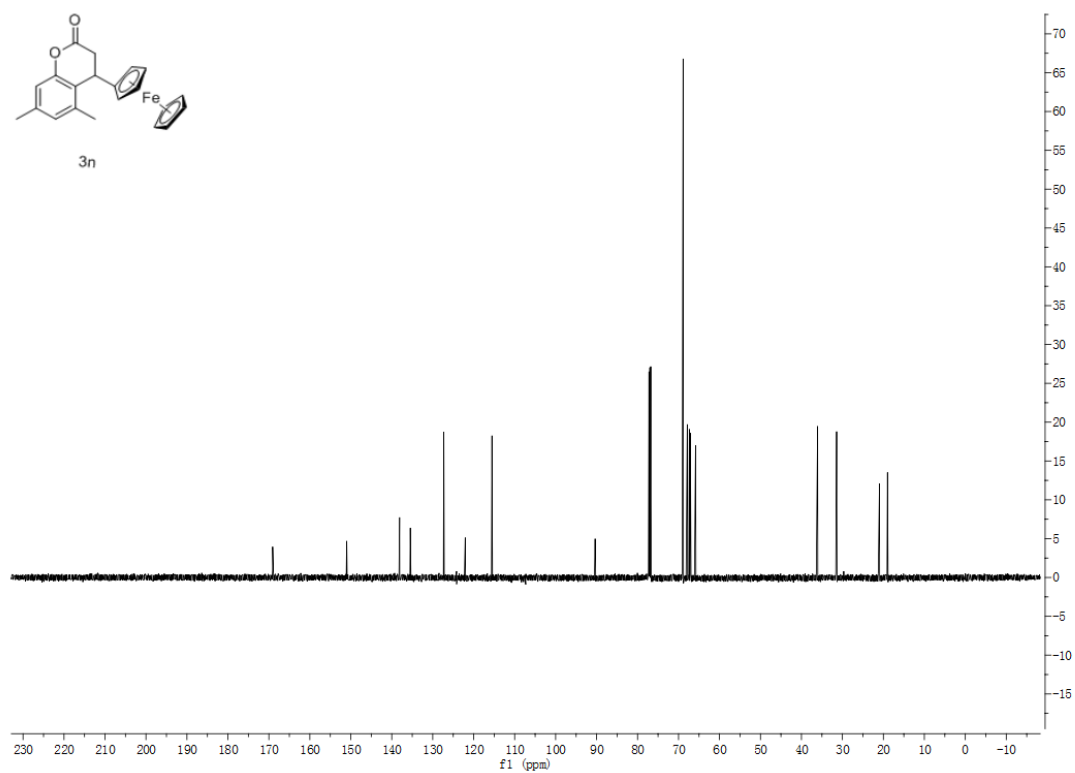

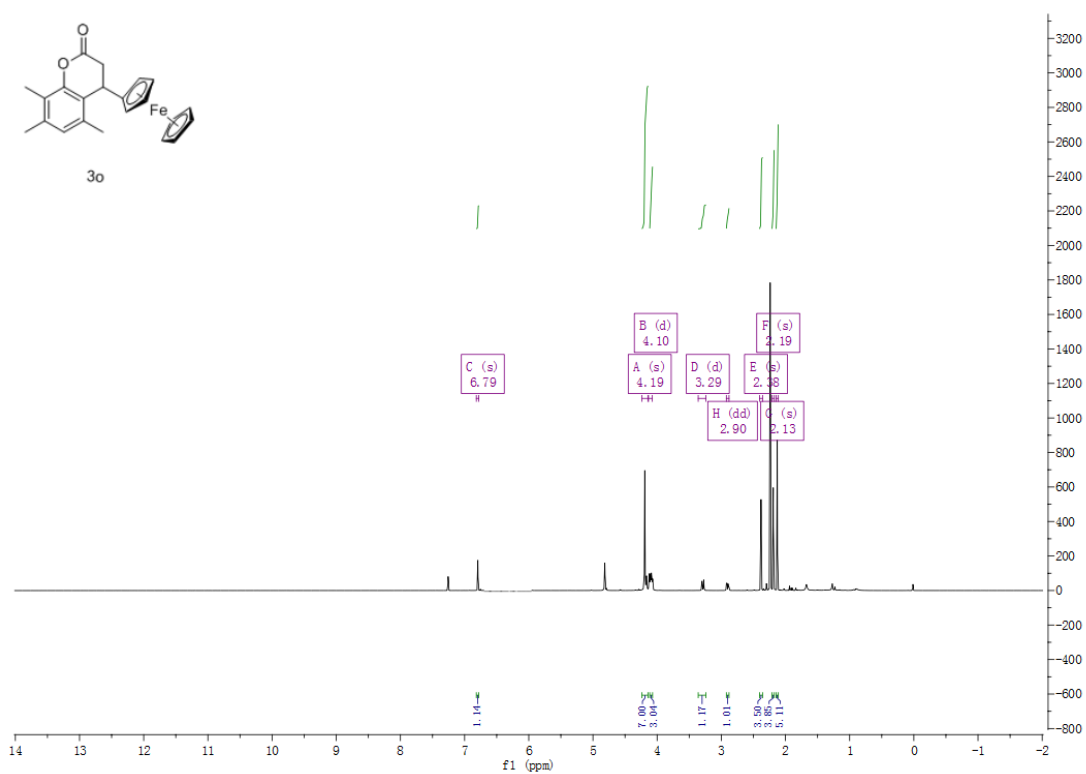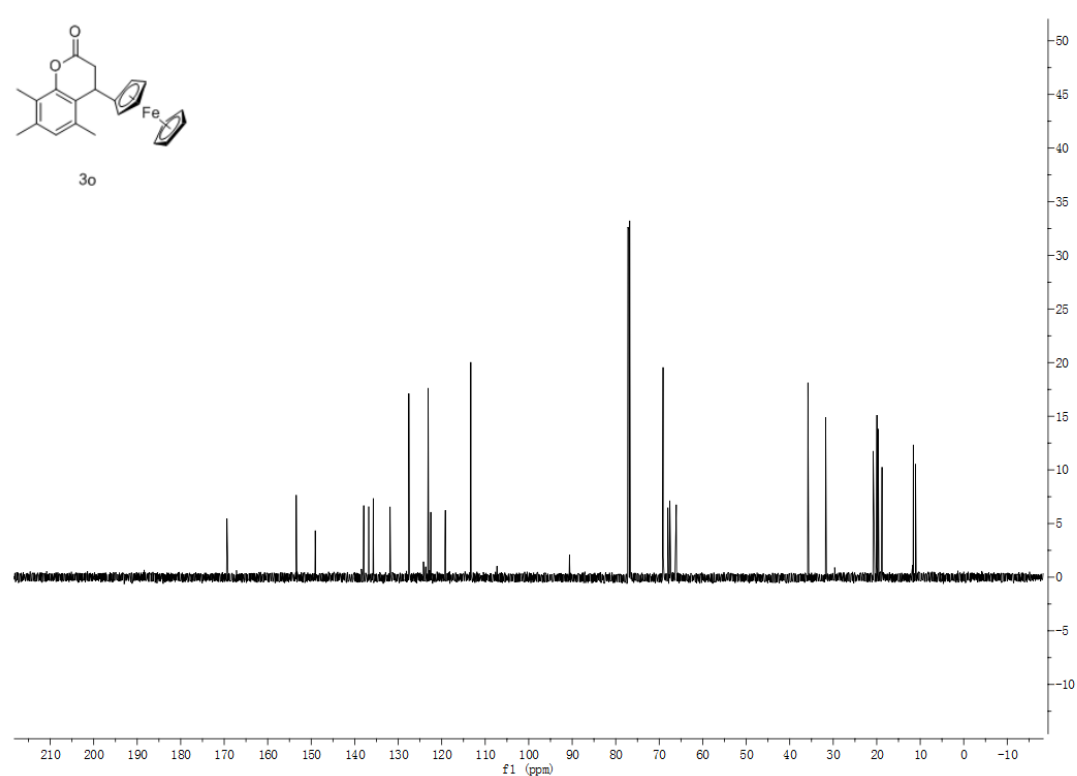

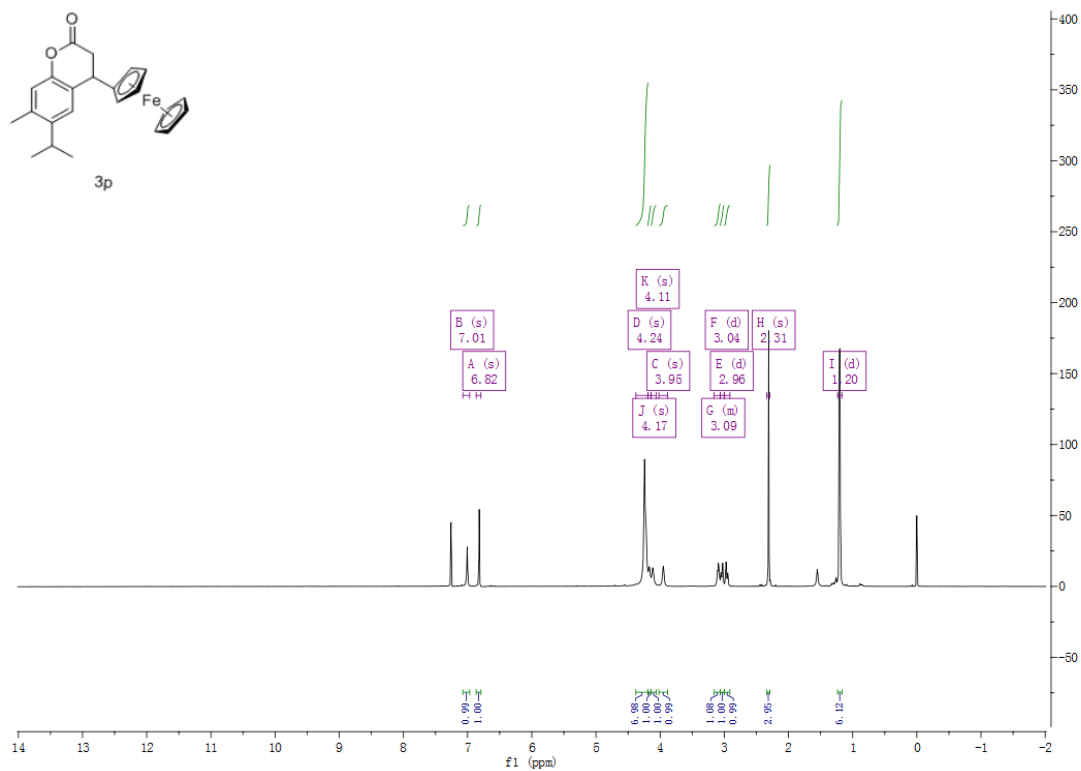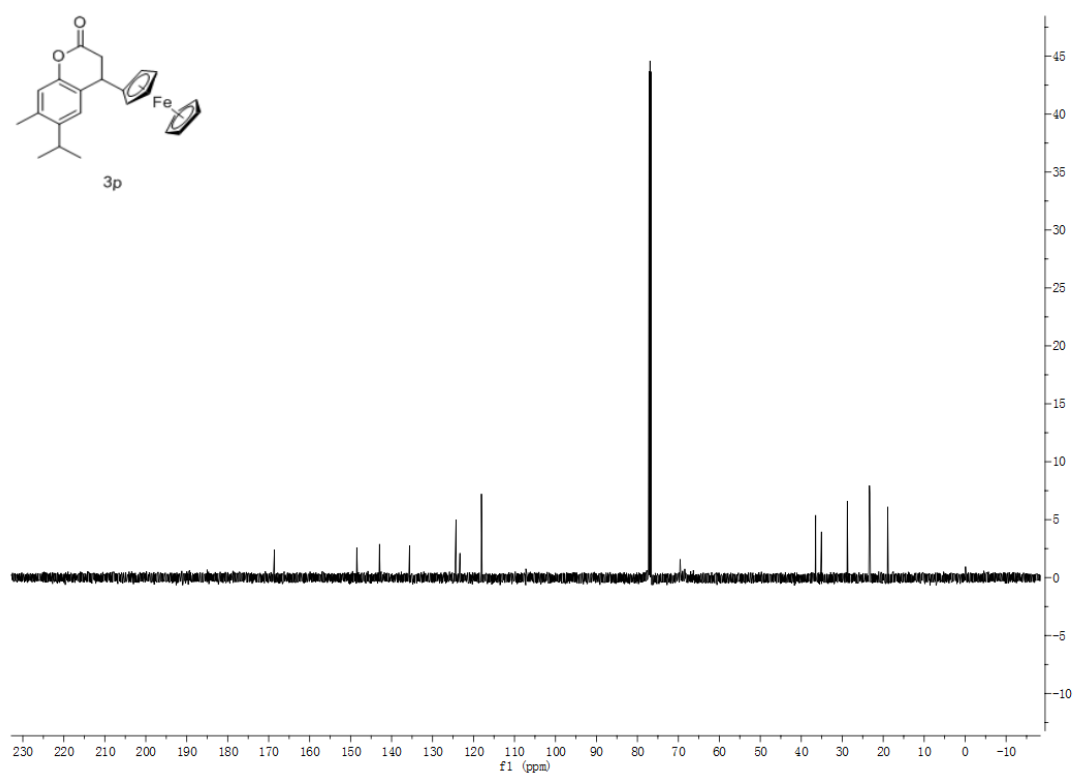

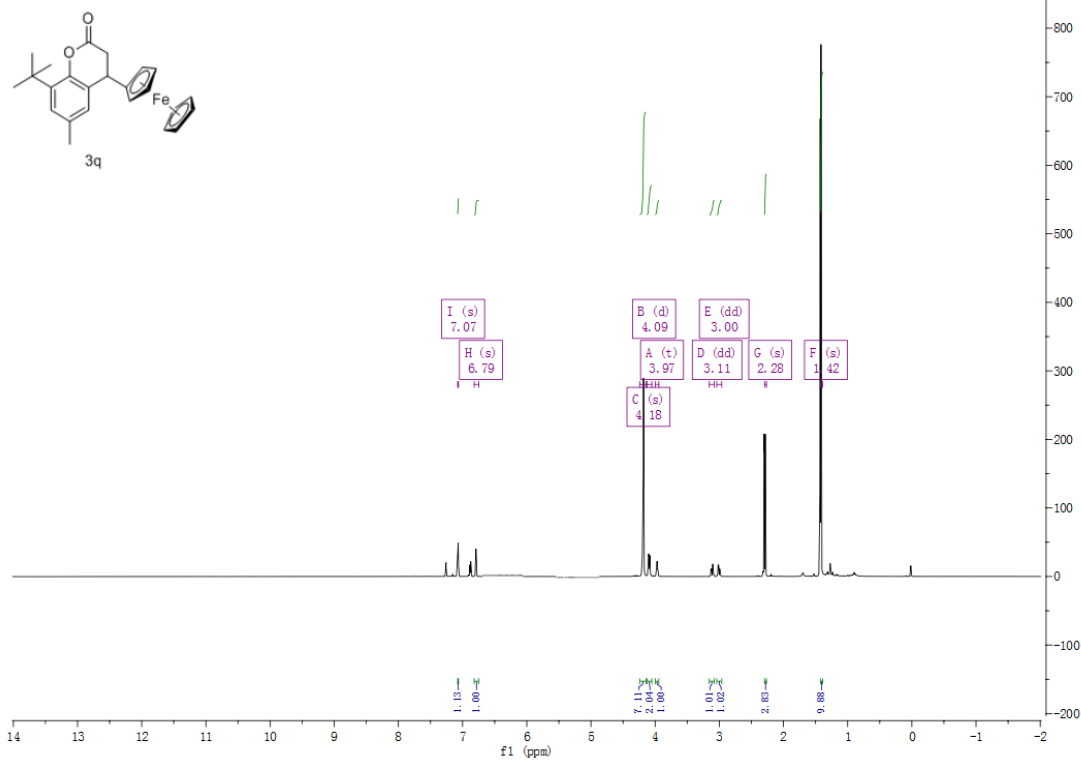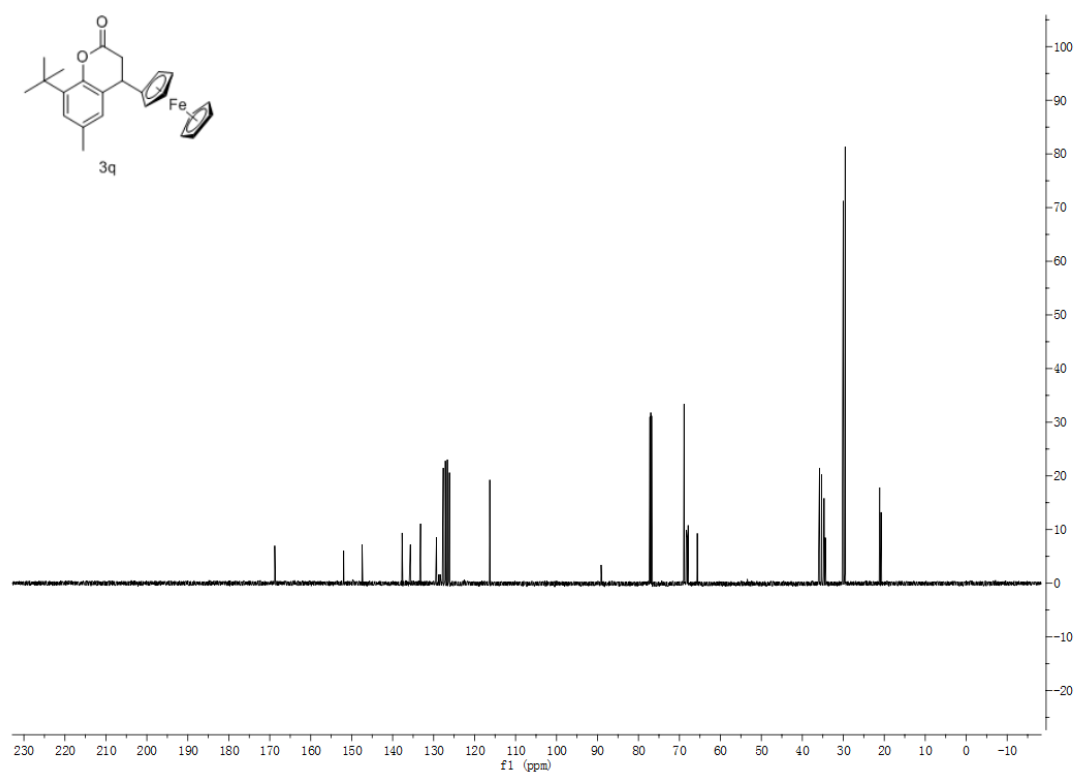

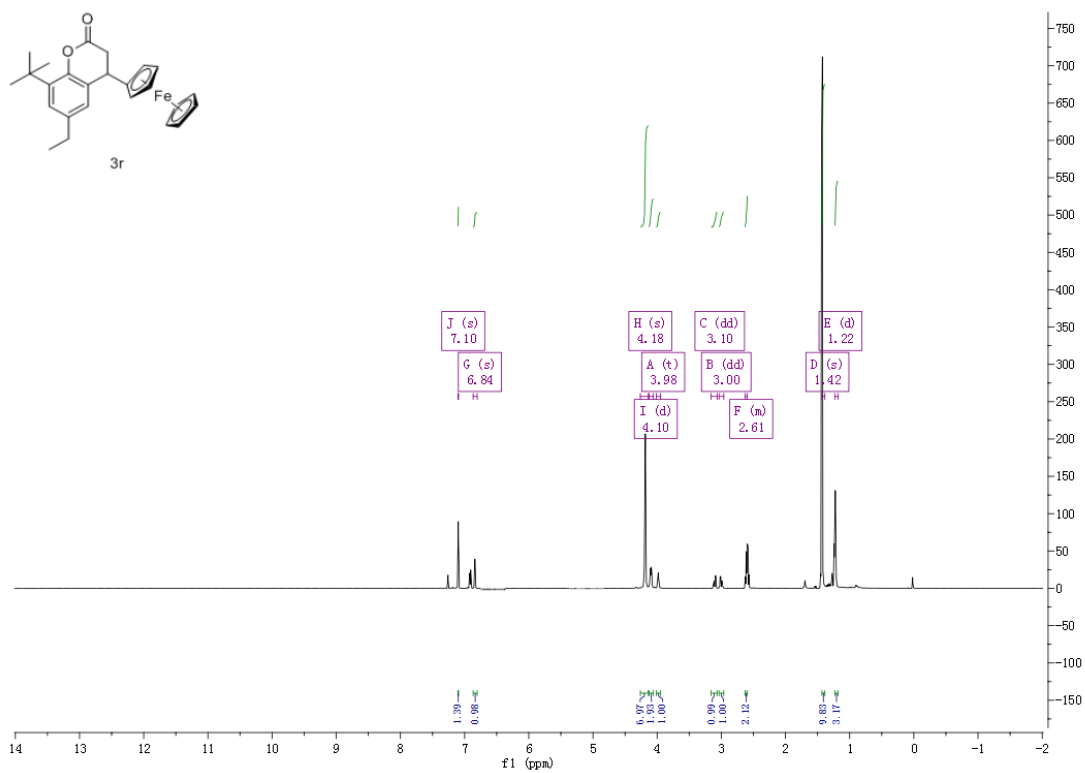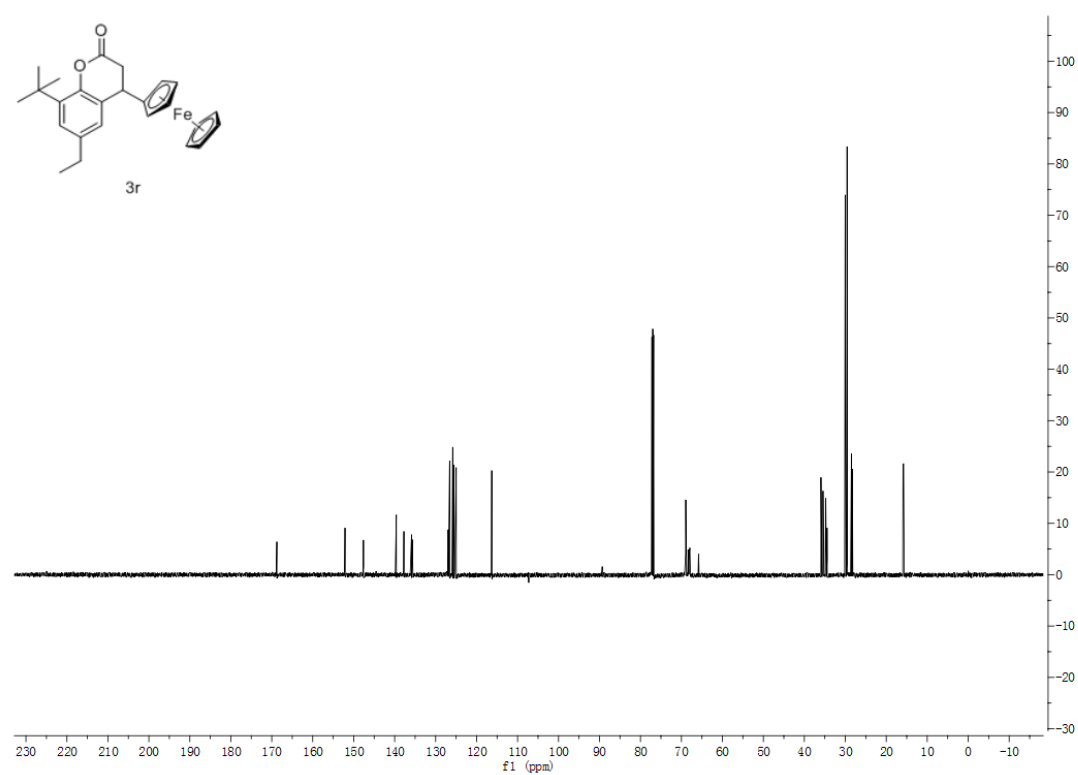

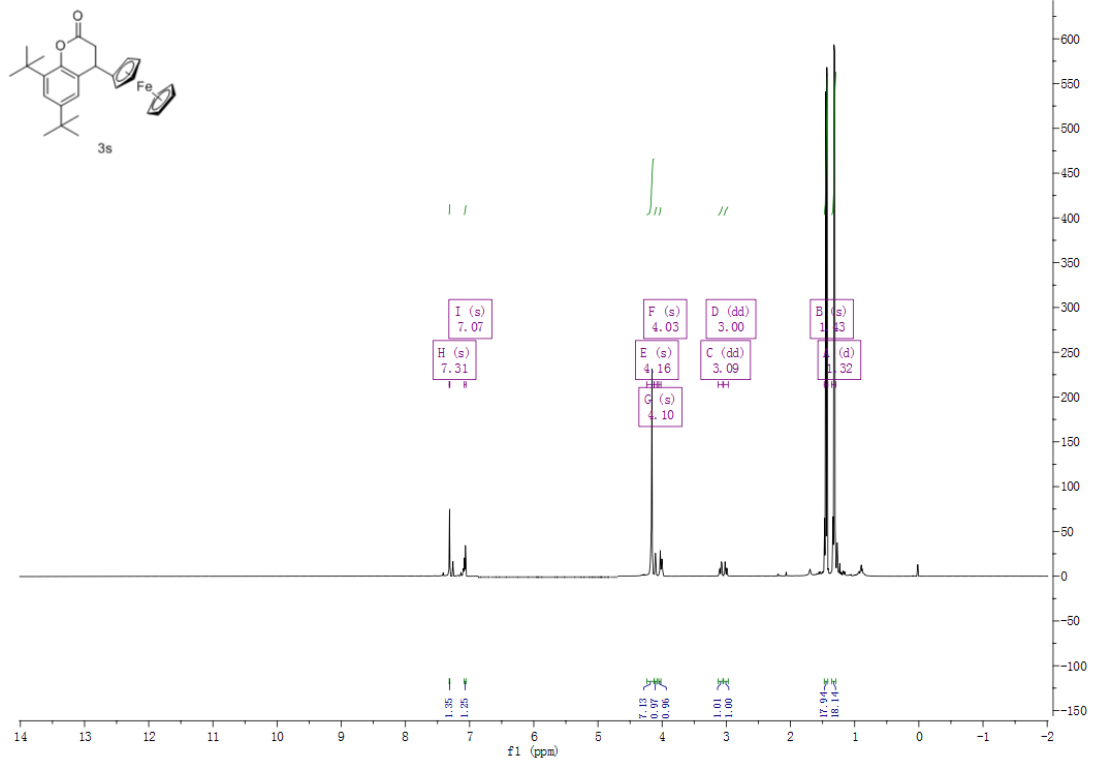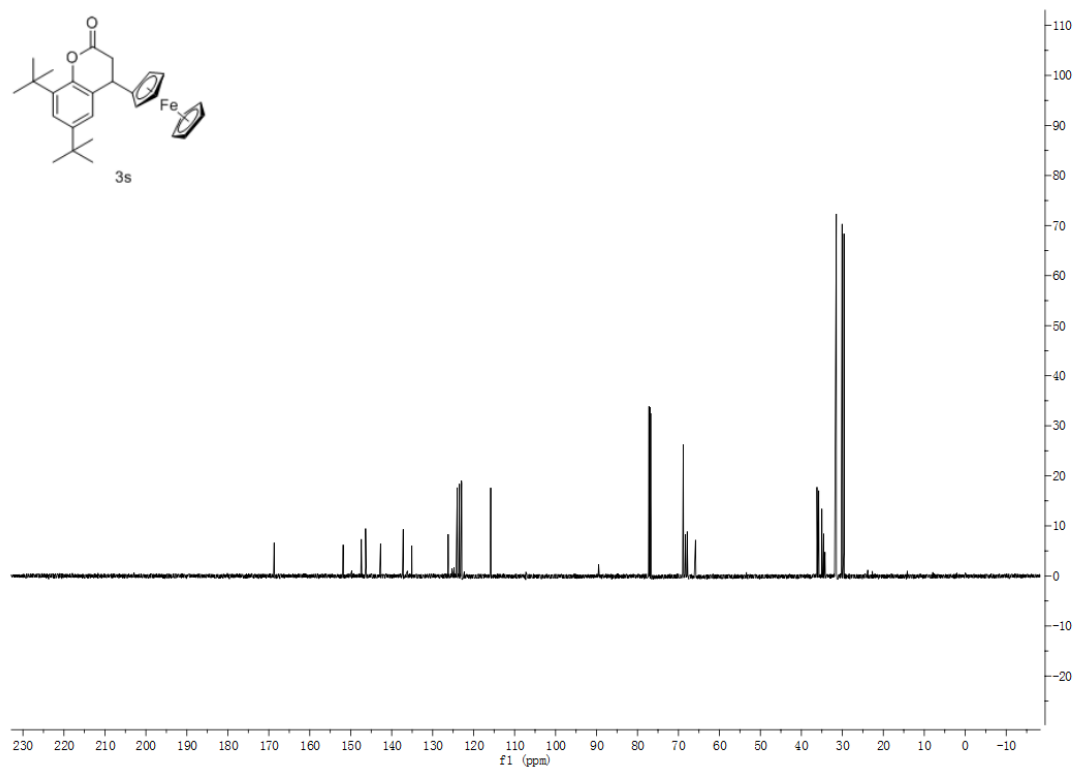

Supplement: Supplemental Material [file IENZ_A_1664499_SM4050.pdf]
